# Human physiologically based pharmacokinetic model for ACE inhibitors: ramipril and ramiprilat

**DOI:** 10.1186/1472-6904-6-1

**Published:** 2006-01-06

**Authors:** David G Levitt, Rik C Schoemaker

**Affiliations:** 1 Department of Physiology, University of Minnesota, 6-125 Jackson Hall, 321 Church St. S. E., Minneapolis, MN 55455, USA; 2 Centre for Human Drug Research Zernikedreef 10, 2333CL Leiden, The Netherlands

## Abstract

**Background:**

The angiotensin-converting enzyme (ACE) inhibitors have complicated and poorly characterized pharmacokinetics. There are two binding sites per ACE (high affinity "C", lower affinity "N") that have sub-nanomolar affinities and dissociation rates of hours. Most inhibitors are given orally in a prodrug form that is systemically converted to the active form. This paper describes the first human physiologically based pharmacokinetic (PBPK) model of this drug class.

**Methods:**

The model was applied to the experimental data of van Griensven et. al for the pharmacokinetics of ramiprilat and its prodrug ramipril. It describes the time course of the inhibition of the N and C ACE sites in plasma and the different tissues. The model includes: 1) two independent ACE binding sites; 2) non-equilibrium time dependent binding; 3) liver and kidney ramipril intracellular uptake, conversion to ramiprilat and extrusion from the cell; 4) intestinal ramipril absorption. The experimental in vitro ramiprilat/ACE binding kinetics at 4°C and 300 mM NaCl were assumed for most of the PBPK calculations. The model was incorporated into the freely distributed PBPK program PKQuest.

**Results:**

The PBPK model provides an accurate description of the individual variation of the plasma ramipril and ramiprilat and the ramiprilat renal clearance following IV ramiprilat and IV and oral ramipril. Summary of model features: Less than 2% of total body ACE is in plasma; 35% of the oral dose is absorbed; 75% of the ramipril metabolism is hepatic and 25% of this is converted to systemic ramiprilat; 100% of renal ramipril metabolism is converted to systemic ramiprilat. The inhibition was long lasting, with 80% of the C site and 33% of the N site inhibited 24 hours following a 2.5 mg oral ramipril dose. The plasma ACE inhibition determined by the standard assay is significantly less than the true in vivo inhibition because of assay dilution.

**Conclusion:**

If the in vitro plasma binding kinetics of the ACE inhibitor for the two binding sites are known, a unique PBPK model description of the Griensven et. al. experimental data can be obtained.

## Background

The angiotensin-converting enzyme (ACE) inhibitors are one of the worst characterized drug classes in terms of their quantitative pharmacokinetics and pharmacodynamics. There are a number of factors that complicate the analysis of this drug class:

1) The ACE enzyme in plasma and tissue has a very high affinity (sub nanomolar) for the ACE inhibitors. This produces extremely non-linear kinetics as the concentration falls from high concentrations when most of the drug is free, to low concentrations when most of the drug is bound to ACE.

2) Although it is known that more than 90% of the total ACE is in the tissues, the quantitative distribution of tissue ACE is not well characterized.

3) Although it is known that ACE has two sites with different inhibitor binding constants, the physiological values of these binding constants are not known.

4) The two ACE binding sites have different catalytic substrate selectivity. Predicting the pharmacodynamics of the ACE inhibitors requires knowledge of these substrate activities.

5) The rate of dissociation of the inhibitor from ACE is so slow (hours) that one cannot assume that there is instantaneous equilibrium between the free and bound inhibitor.

6) Assays of the ACE inhibition are unreliable because of uncertainties about the relationship between the in vivo inhibition and the inhibition measured in the standard ACE assay.

7) Most ACE inhibitors are administered orally in the form of a prodrug that is systemically converted to the active inhibitor. Prediction of the pharmacokinetics of the active form requires an understanding of the pharmacokinetics of the prodrug and drug and the details of the conversion of the prodrug to the active form in the liver and kidney.

This paper presents the first attempt to describe a quantitative human physiologically based pharmacokinetic model (PBPK) of the ACE inhibitors. The model describes the pharmacokinetics in terms of realistic human parameters such as the organ blood flows, tissue cellular and extracellular volume and cell membrane permeability. The model incorporates all of the complexities listed above. It is implemented in PKQuest, a general pharmacokinetic software routine that has now been applied to more than 25 different solutes with a wide range of pharmacokinetic properties [1-8]. Many of the physiological parameters of the model have been determined previously by application of PKQuest to other drugs and are used directly without modification in this ACE inhibitor model. This includes the tissue blood flows, extracellular volume of distribution of the different tissues, and the tissue albumin concentration (which is important because of non-specific ACE inhibitor albumin binding).

Although it is now generally recognized that rational drug therapy should be based on the quantitative degree on tissue ACE inhibition [9], the dependence of the pharmacodynamic effect on plasma drug levels and ACE activity is still not clear [10-12]. Huge efforts have been directed at determining optimum dosage with limited results [13]. It will be shown that this PBPK model has the potential to provide direct quantitative information about the time and dose dependence of the ACE inhibition in the different tissues.

It has been known for many years that inhibitor binding to ACE was of the "slow tight" class, with dissociation constants in the sub-nanomolar range, and time constants for release from the tight complex of many hours. Because of the non-linear binding curves, it was initially assumed that the binding of the inhibitor (R) to the free enzyme (E) was a two step process:

R+E↔REL⇄RET     (1)
 MathType@MTEF@5@5@+=feaafiart1ev1aaatCvAUfeBSjuyZL2yd9gzLbvyNv2CaerbwvMCKfMBHbqedmvETj2BSbqee0evGueE0jxyaibaieYdOi=BH8vipeYdI8qiW7rqqrFfpeea0xe9Lq=Jc9vqaqpepm0xbbG8FasPYRqj0=yi0lXdbba9pGe9qqFf0dXdHuk9fr=xfr=xfrpiWZqaaeaabiGaaiaacaqabeaabeqacmaaaOqaaiaadkfacqGHRaWkcaWGfbGaeyiLHSQaamOuamaaBaaaleaacaWGfbGaamitaaqabaGccqWIehcGcaWGsbWaaSbaaSqaaiaadweacaWGubaabeaakiaaxMaacaWLjaWaaeWaaeaacaaIXaaacaGLOaGaayzkaaaaaa@3F07@

with rapid formation of a loose complex (R_EL_) followed by a slow conversion to the tight form (R_ET_) [14-18]. When it was recognized that ACE had two homologous extracellular binding sites (referred to as the "N" and "C" terminal sites), it became apparent that the non-linearity resulted from the presence of these two sites and that the kinetics of each site could be described as a one step process characterized by two equilibrium dissociation constants K_N _and K_C _(nM) and unbinding rate constants k_-N _and k_-C _(1/min) [19]:

R+EC⇄k−CkCRECKC=k−C/kCR+EN⇄k−NkNRENKN=k−N/kN     (2)
 MathType@MTEF@5@5@+=feaafiart1ev1aaatCvAUfKttLearuWrP9MDH5MBPbIqV92AaeXatLxBI9gBaebbnrfifHhDYfgasaacH8akY=wiFfYdH8Gipec8Eeeu0xXdbba9frFj0=OqFfea0dXdd9vqai=hGuQ8kuc9pgc9s8qqaq=dirpe0xb9q8qiLsFr0=vr0=vr0dc8meaabaqaciaacaGaaeqabaqabeGadaaakeaafaqaaeGacaaabaGaemOuaiLaey4kaSIaemyrau0aaSbaaSqaaiabdoeadbqabaGcdaGdnaWcbaGaem4AaS2aaSbaaWqaaiabdoeadbqabaaaleaacqWGRbWAdaWgaaadbaGaeyOeI0Iaem4qameabeaaaOGaayPKHiaawcziaiabdkfasnaaBaaaleaacqWGfbqrcqWGdbWqaeqaaaGcbaGaem4saS0aaSbaaSqaaiabdoeadbqabaGccqGH9aqpcqWGRbWAdaWgaaWcbaGaeyOeI0Iaem4qameabeaakiabc+caViabdUgaRnaaBaaaleaacqWGdbWqaeqaaaGcbaGaemOuaiLaey4kaSIaemyrau0aaSbaaSqaaiabd6eaobqabaGcdaGdnaWcbaGaem4AaS2aaSbaaWqaaiabd6eaobqabaaaleaacqWGRbWAdaWgaaadbaGaeyOeI0IaemOta4eabeaaaOGaayPKHiaawcziaiabdkfasnaaBaaaleaacqWGfbqrcqWGobGtaeqaaaGcbaGaem4saS0aaSbaaSqaaiabd6eaobqabaGccqGH9aqpcqWGRbWAdaWgaaWcbaGaeyOeI0IaemOta4eabeaakiabc+caViabdUgaRnaaBaaaleaacqWGobGtaeqaaaaakiaaxMaacaWLjaWaaeWaaeaacqaIYaGmaiaawIcacaGLPaaaaaa@67E4@

There is conflicting data about the interaction between the two sites (see [20] for recent discussion). In the most detailed experimental measurements of the ACE binding kinetics, Wei et. al. [19] found that the two sites acted independently in the binding of [^3^H]trandolaprilat. It is assumed here that the N and C site binding of ramiprilat is also independent.

The only quantitative kinetic measurements of K_i _and k_-i _for ramiprilat binding to the two sites is at 4°C and 300 mM NaCl [21]. Measurements of the rate of substrate hydrolysis indicates that, in going from 25°C and 300 mM Cl^- ^to 37°C and 120 mM Cl^-^, the change in apparent K_i _of ramiprilat is small because the temperature and chloride change tend to cancel each other [22]. In the following analysis, as a first approximation, it will be assumed that the physiological (37°C and 100 mM Cl) binding constants for the two sites (eq. (2)) are equal to the experimental values at 4°C and 300 mM NaCl.

Although there have been some reports of different binding properties of plasma and tissue ACE [23-27], the differences are small and could be an artifact of the difficulty of assaying these very high affinity ("tight") enzymes (see below). Since it has been established that the circulating ACE in the plasma is derived from the membrane bound tissue ACE by post-translational proteolytic cleavage [28,29], it will be assumed, as a first approximation, that the circulating and tissue ACE are identical. (A major exception is testis ACE which contains only the C terminal domain binding site [21].) This is an important consideration because it allows one to use measurements of the fraction of the circulating plasma ACE that is occupied by bound inhibitor to determine the ACE inhibition in the different tissues – the clinically important factor.

Previous attempts to model the pharmacokinetics and pharmacodynamics of the ACE inhibitors have used compartmental models and were not physiologically based (i.e., using known organ blood flows, etc.) [30-34]. Toutain and colleagues have developed detailed compartment models and used them to accurately describe the non-linear pharmacokinetics/pharmacodynamics of a number of high affinity ACE inhibitors in animals [30-32]. The most important previous modeling of ramiprilat in humans is that of Brockmeier [35,36] who recognized that the renal clearance of ramiprilat provided a direct measurement of the fraction that was free in plasma and used this measurement to estimate the physiological ramiprilat ACE binding constant. All of these earlier models have been limited by the assumption of a single ACE binding site and equilibrium binding and they have not attempted to model the processes involved in the conversion of the prodrug to the drug.

**Figure 1:**
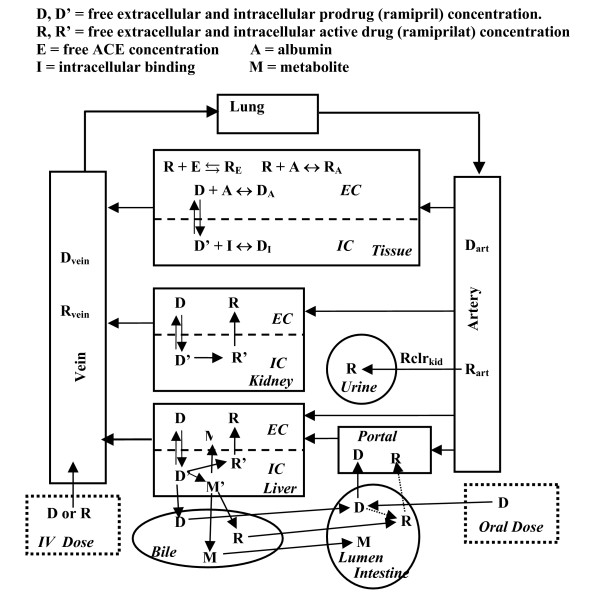
PBPK model of the pharmacokinetics of prodrug ramipril (R) and active drug ramiprilat (D). The figure schematically illustrates the events in three different tissues (typical "tissue", the liver and the kidney), the intestinal absorption, the renal clearance and enterohepatic recirculation.

The PBPK model that is applied here to the ACE inhibitors is summarized in fig. 1. It has two features that are implemented for the first time in a PBPK model. The first is the use of a time dependent binding (eq. (2)) in each of the tissues, in place of the usual assumption of equilibrium binding. The second is the physiological model of the cellular uptake and intracellular conversion of ramipril to ramiprilat and the subsequent extrusion to the circulating blood. These features have been implemented in the freely distributed software program PKQuest [37].

The model is applied to the detailed pharmacokinetic analysis of ramipril (prodrug) and ramiprilat (the active dicarboxylic acid ACE inhibitor) previously described by van Griensven et. al[38]. This is an open, randomized, three-way cross-over study that measured: 1) the plasma ramiprilat following an intravenous (IV) ramiprilat infusion; 2) plasma ramipril and ramiprilat following an IV ramipril infusion; and 3) plasma ramipril and ramiprilat after oral ramipril. In addition, plasma ACE activity and the urinary excretion of ramipril, ramiprilat and the major metabolites were measured in all 3 arms. The requirement that a single PBPK model must be able to describe all these data sets places strong constraints on the model and severely limits the range of the allowed model parameters.

## Methods

### Notation

R, D – free, unbound, concentration of ramipril and ramiprilat, respectively.

k_N_, k_C_, k_-N_, k_-C _– association (1/(nM min)) and dissociation rate (1/min) rate constants for N terminal and C terminal ACE binding site.

K_N_, K_C _– equilibrium disassociation constant for N terminal and C terminal ACE binding site.

Cl – plasma clearance in terms of total plasma concentration.

Cl_u_, Cl_R _– free (unbound) and total arterial kidney ramiprilat clearance.

Cl_int_L_, Cl_int_K _– intrinsic liver and kidney clearance of ramipril in terms of free, unbound tissue concentration.

f_u_, f_u_cell _– fraction unbound in plasma and in intracellular water.

V_w _– intracellular water volume.

P, S, Ps – Cell membrane permeability, surface area, and permeability coefficient (Ps = PS/V_w_)

A_D_, a, T – gamma function parameters describing intestinal absorption of ramipril (A_D _= total amount absorbed.).

A_R_, A_slow _– amount of intestinal absorption in the form of ramiprilat and the slow ramipril absorption component.

### The PBPK model

**Figure 2:**
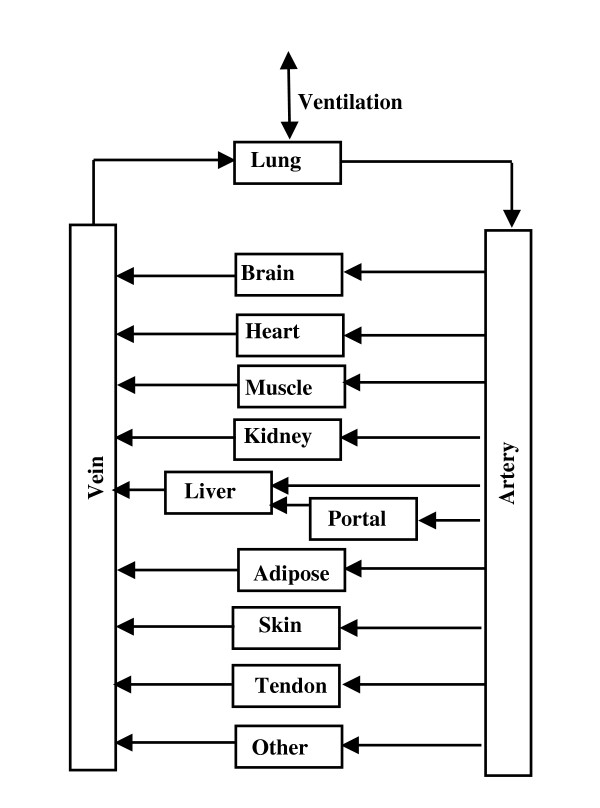
Schematic diagram of the arrangement of the different tissues in the PBPK model. The organ "portal" refers to all the organs drained by the portal vein. The connective tissue is divided between two organs: "tendon" with a relatively low blood flow and "other" with a higher blood flow.

This section describes the main features of the model. See additional file ACE_supplemental_31oct05.doc (section I) for a detailed mathematical description. The arrangement of the different tissues is shown in fig. 2. The tissue parameters (blood flow, volumes, etc.) are listed in Table 1 and are identical to those used in previous applications of PKQuest [1-8]. The connective tissue is divided between two organs: "tendon" with a relatively low blood flow, and "other" with a higher blood flow. The "standard" organ blood flows are assumed and the small changes in peripheral blood flow produced by ramipril [39] are neglected. ACE inhibitors do not produce a significant change in cardiac output [40].

**Table 1:** Standard human population PBPK organ parameters (not varied).

**Organ**	**Weight (Kg)**	**ECF water(L)**	**Flow (L/min)**	**ACE Tiss/Plasma**	**Ramiprialat Ps (min^-1^)**	**Ramiprilat fu_cell**	**Ramipril Ps (min^-1^)**	**Ramipril fu_cell**
**Blood**	5.5	2.68345		0.52	0	----		
**liver**	1.8	0.2898	0.45	1.37	Adjustable	1	infinite	0.1
**portal **	1.5	0.351	1.125	2.0	0	----	0.05	0.5
**muscle**	26	3.042	0.585	1.0	0	----	0.05	0.5
**kidney**	0.31	0.04092	1.24	5.62	Adjustable	10	infinite	0.01
**brain**	1.4	0	0.784	0.8	0	----	0.05	0.5
**heart**	0.33	0.066	0.264	1.7	0	----	0.05	0.5
**lung**	0.536	0.08576	5.6184	34	0	----	0.05	0.5
**skin**	2.6	1.092	0.26	3.51	0	----	0.05	0.5
**tendon**	3	2.55	0.03	7.23	0	----	0.05	0.5
**other**	5.524	3.75632	0.1104	5.79	0	----	0.05	0.5
**bone**	4	0	0	0	0	----	0	0.5
**adipose**	17.5	3.5	0.7385	6.81	0	----	0.05	0.5

**Total**	70	17.4572	5.5877					

Figure 1 shows a more detailed view of the model that focuses on 3 tissues: the liver, kidney, and one representative "tissue" region. "D" is the free prodrug (ramipril) concentration, and "R" is the free active drug (ramiprilat) concentration. The top square, labeled "tissue" shows the kinetic processes in a typical tissue region. Both the highly water soluble ramiprilat (R) and the tissue ACE binding sites are restricted to the extracellular space (EC). (Exceptions are the liver and kidney which have ramiprilat cell membrane transport systems.) The fraction of tissue volume that is extracellular has been determined previously [6]. As described in eq. 2, it is assumed that ACE has two non-interacting binding sites (labeled as "C" and "N" terminal) with different ramiprilat binding constants [19,21] (only one binding site is shown in fig. 1). The binding for each site is characterized by the two parameters: k_-i _the unbinding rate constant; and K_i _the dissociation equilibrium constant (k_i _= k_-i_/K_i_). The amount bound is described quantitatively by the differential equations:

d(REC)dt=kCR EC−k−CRECd(REN)dt=kNR EN−k−NREN     (3)
 MathType@MTEF@5@5@+=feaafiart1ev1aaatCvAUfKttLearuWrP9MDH5MBPbIqV92AaeXatLxBI9gBaebbnrfifHhDYfgasaacH8akY=wiFfYdH8Gipec8Eeeu0xXdbba9frFj0=OqFfea0dXdd9vqai=hGuQ8kuc9pgc9s8qqaq=dirpe0xb9q8qiLsFr0=vr0=vr0dc8meaabaqaciaacaGaaeqabaqabeGadaaakeaafaqabeqacaaabaWaaSaaaeaacqWGKbazcqGGOaakcqWGsbGudaWgaaWcbaGaemyrauKaem4qameabeaakiabcMcaPaqaaiabdsgaKjabdsha0baacqGH9aqpcqWGRbWAdaWgaaWcbaGaem4qameabeaakiabdkfasjaaykW7cqWGfbqrdaWgaaWcbaGaem4qameabeaakiabgkHiTiabdUgaRnaaBaaaleaacqGHsislcqWGdbWqaeqaaOGaemOuai1aaSbaaSqaaiabdweafjabdoeadbqabaaakeaadaWcaaqaaiabdsgaKjabcIcaOiabdkfasnaaBaaaleaacqWGfbqrcqWGobGtaeqaaOGaeiykaKcabaGaemizaqMaemiDaqhaaiabg2da9iabdUgaRnaaBaaaleaacqWGobGtaeqaaOGaemOuaiLaaGPaVlabdweafnaaBaaaleaacqWGobGtaeqaaOGaeyOeI0Iaem4AaS2aaSbaaSqaaiabgkHiTiabd6eaobqabaGccqWGsbGudaWgaaWcbaGaemyrauKaemOta4eabeaaaaGccaWLjaGaaCzcamaabmaabaGaeG4mamdacaGLOaGaayzkaaaaaa@6516@

There is also a non-specific, non-saturating equilibrium ACE inhibitor binding to the plasma and tissue albumin (A) characterized by the albumin binding constant (B_R_):

R+A↔BRRARA=(BRA) R     (4)
 MathType@MTEF@5@5@+=feaafiart1ev1aaatCvAUfKttLearuWrP9MDH5MBPbIqV92AaeXatLxBI9gBaebbnrfifHhDYfgasaacH8akY=wiFfYdH8Gipec8Eeeu0xXdbba9frFj0=OqFfea0dXdd9vqai=hGuQ8kuc9pgc9s8qqaq=dirpe0xb9q8qiLsFr0=vr0=vr0dc8meaabaqaciaacaGaaeqabaqabeGadaaakeaafaqabeqacaaabaGaemOuaiLaey4kaSIaemyqae0aa4anaSqaaiabdkeacnaaBaaameaacqWGsbGuaeqaaaWcbeGccaGLugcacqWGsbGudaWgaaWcbaGaemyqaeeabeaaaOqaaiabdkfasnaaBaaaleaacqWGbbqqaeqaaOGaeyypa0JaeiikaGIaemOqai0aaSbaaSqaaiabdkfasbqabaGccqWGbbqqcqGGPaqkcaaMc8UaemOuaifaaiaaxMaacaWLjaWaaeWaaeaacqaI0aanaiaawIcacaGLPaaaaaa@4579@

As describe in the additional file 1 ACE_supplemental_31oct05.doc (section I), the amount of albumin binding in the different tissues can be determined from the experimental value for the fraction of albumin bound ramiprilat in plasma (= 0.56 [41]) and the previously determined "standard human" PKQuest values for the tissue extracellular albumin [6]. This supplemental file section also describes in more detail how the above relations for ramiprilat are incorporated into the PKQuest PBPK routine.

The PBPK analysis indicates that ramipril has a finite cell membrane permeability that allows it to enter the intracellular space (IC) (see fig. 1, "tissue"). This is consistent with the relatively high octanol/water partition coefficient at pH 7 of 1.12 of ramipril, one hundred fold greater than that of ramiprilat (0.011) [42]. The rate of exchange between the intracellular and extracellular space is determined by two parameters: 1) the cell membrane permeability coefficient (Ps); and 2) the fraction of intracellular solute that is unbound (f_u_cell_):

d(D')dt=fu_cellPs(D−D')Ps=PS/Vw     (5)
 MathType@MTEF@5@5@+=feaafiart1ev1aaatCvAUfKttLearuWrP9MDH5MBPbIqV92AaeXatLxBI9gBaebbnrfifHhDYfgasaacH8akY=wiFfYdH8Gipec8Eeeu0xXdbba9frFj0=OqFfea0dXdd9vqai=hGuQ8kuc9pgc9s8qqaq=dirpe0xb9q8qiLsFr0=vr0=vr0dc8meaabaqaciaacaGaaeqabaqabeGadaaakeaafaqabeqacaaabaWaaSaaaeaacqWGKbazcqGGOaakcqWGebarcqGGNaWjcqGGPaqkaeaacqWGKbazcqWG0baDaaGaeyypa0JaemOzay2aaSbaaSqaaiabdwha1jabc+faFjabdogaJjabdwgaLjabdYgaSjabdYgaSbqabaGccqWGqbaucqWGZbWCcqGGOaakcqWGebarcqGHsislcqWGebarcqGGNaWjcqGGPaqkaeaacqWGqbaucqWGZbWCcqGH9aqpcqWGqbaucqWGtbWucqGGVaWlcqWGwbGvdaWgaaWcbaGaem4DaChabeaaaaGccaWLjaGaaCzcamaabmaabaGaeGynaudacaGLOaGaayzkaaaaaa@54D5@

where D and D' are the free extracellular and intracellular ramipril concentration and Ps is the cellular permeability coefficient (P = permeability, S = surface area, and V_w _= intracellular water volume). Extracellular ramipril is also bound to albumin, similar to the binding of ramiprilat (eq. (4)).

The ramiprilat and ramipril kinetics discussed above and illustrated in fig. 1 for the box labeled "tissue" is present in all the tissues, including the liver and kidney and central venous and arterial compartment (binding not shown in fig. 1). Each tissue i is characterized by 3 parameters: 1) total ACE concentration (ACE_i_) with two sites per each ACE molecule (Et_C_^i ^= Et_N_^i ^= ACE_i_); 2) ramipril cell membrane permeability coefficient (Ps_i_) and 3) intracellular ramipril binding (f_u_cell_). In addition, the ramiprilat liver and kidney Ps and f_u_cell _must be defined. The ramiprilat ACE binding constants (K_N_, K_C_, k_-N_, k_-C_) are assumed to be identical for all tissues.

Both the liver and kidney metabolize the intracellular ramipril (D'). As shown in fig. 1, the liver metabolizes ramipril to ramiprilat (R') and to a number of other metabolites (indicated by M). Three parameters characterize this metabolism: the intrinsic liver (Cl_int_L_) and kidney (Cl_int_K_) clearance and the fraction of total liver ramipril metabolism that is converted to ramiprilat (Fr_L_). (1- Fr_L _is converted to other metabolites). It is assume that all the ramipril metabolized by the kidney is converted to ramiprilat. The rate that the liver converts intracellular ramipril (free concentration = D') to intracellular ramiprilat (R') is:

*Q_R _= Cl*_int_*L*_*Fr_L _D*'     (6)

All the intracellular ramiprilat generated by the liver and kidney eventually enters the systemic circulation (see eq. (5)). The intrinsic clearance is in terms of the free concentration. For a flow limited, well stirred tissue, the absolute clearance (Cl) can be approximately related to the intrinsic clearance (Cl_int_) in terms of the organ flow (F), and fraction unbound (f_u_):

*Cl = f_u _Cl*_int _*F*/(*F + f_u _Cl*_int_)     (7)

As illustrated in fig. 1, the extracellular ramiprilat is not metabolized or excreted by the liver. The only excretory pathway removing ramiprilat is the renal excretion (Q_kidney_) which is assumed to be proportional to the free, unbound, arterial ramiprilat concentration (R_art_):

*Q_kidney _= Cl_u _R_art _*    (8)

Where Cl_u _is the unbound renal clearance. The unbound concentration is related to the total arterial plasma concentration (R_T_art_) by the fraction unbound in arterial plasma (f_u_):

*Q_kidney _= Cl_u _R_art _= Cl_u _f_u_R_T_art _= Cl_R_R_T_art _    Cl_R _= f_u_Cl_u _*    (9)

where Cl_R _is the renal total plasma clearance. Because of the highly non-linear ramiprilat binding that results from saturation of the ACE binding sites, the total renal clearance (Cl_R_) is non-linear: at high concentrations when most of the ramiprilat is free (f_u_→ 1), the clearance is equal to the intrinsic unbound clearance (Cl_R _≈ Cl_u _≈ 0.4 l/min), while at long times and low concentrations, when most of the ramiprilat is bound, the clearance falls to values less than 0.02 l/min [35,36].

The observed delay in the systemic appearance of ramiprilat after either an oral or IV ramipril input depends on the processes involved in the liver and kidney ramipril cellular uptake, intracellular conversion to ramiprilat and then transport to the plasma. These processes have been directly identified in an extensive series of investigations of Pang and colleagues on the behavior of enalapril and enalaprilat in perfused rat liver, kidney and intestine [43-49].

Although there was an initial report that the intestinal absorption was carrier mediated [50], Morrison et. al. [51] have clearly shown that the absorption of enalapril is primarily a non-saturable, passive diffusion process. There are three different components for absorption of oral ramipril (fig. 1). The major component is direct absorption of ramipril into the portal circulation where it is subject to first pass metabolism. This component is described by the 3 parameter gamma distribution (A_D_, T, a):

*I*(*t*) = *A_D_b^a^t*^*a*-1 ^exp(-*bt*)/Γ(*a*)     *b = a*/*T *    (10)

where A_D _is the total amount absorbed, Γ is the gamma function, a is the dimensionless gamma number and T is a time constant. For most drugs this 3 parameter function provides a good description of the time delay in oral absorption produced by gastric emptying [5]. The three parameters A_D_, a and T are adjusted for each subject. Pang et. al. [48] have shown in the rat that there is a second component in which some of the oral ramipril can be directly converted to ramiprilat either in the lumen or the intestinal epithelial cells and absorbed into the portal blood. This second component produces a more rapid systemic appearance of ramiprilat than the first component because it bypasses the slower processes involved in the liver and renal production of ramiprilat from ramipril. It is assumed that this ramiprilat absorption has the same time course as the major ramipril component (eq. (10)) with the same values of a and T and a value of A (= A_R_) about 10% that of A_D_. Finally, in some subjects it was necessary to add a second, much slower rate of ramipril absorption that was described by a constant rate that extended from 200 to 3000 minutes:

I(t)={0t<200Io200<t<30000t>3000}I0=Aslow/(3000−200)     (11)
 MathType@MTEF@5@5@+=feaafiart1ev1aaatCvAUfKttLearuWrP9MDH5MBPbIqV92AaeXatLxBI9gBaebbnrfifHhDYfgasaacH8akY=wiFfYdH8Gipec8Eeeu0xXdbba9frFj0=OqFfea0dXdd9vqai=hGuQ8kuc9pgc9s8qqaq=dirpe0xb9q8qiLsFr0=vr0=vr0dc8meaabaqaciaacaGaaeqabaqabeGadaaakeaafaqabeqacaaabaGaemysaKKaeiikaGIaemiDaqNaeiykaKIaeyypa0ZaaiWaaeaafaqabeWacaaabaGaeGimaadabaGaemiDaqNaeyipaWJaeGOmaiJaeGimaaJaeGimaadabaGaemysaK0aaSbaaSqaaiabd+gaVbqabaaakeaacqaIYaGmcqaIWaamcqaIWaamcqGH8aapcqWG0baDcqGH8aapcqaIZaWmcqaIWaamcqaIWaamcqaIWaamaeaacqaIWaamaeaacqWG0baDcqGH+aGpcqaIZaWmcqaIWaamcqaIWaamcqaIWaamaaaacaGL7bGaayzFaaaabaGaemysaK0aaSbaaSqaaiabicdaWaqabaGccqGH9aqpcqWGbbqqdaWgaaWcbaGaem4CamNaemiBaWMaem4Ba8Maem4DaChabeaakiabc+caViabcIcaOiabiodaZiabicdaWiabicdaWiabicdaWiabgkHiTiabikdaYiabicdaWiabicdaWiabcMcaPaaacaWLjaGaaCzcamaabmaabaGaeGymaeJaeGymaedacaGLOaGaayzkaaaaaa@6746@

where A_slow _is the total amount absorbed by this component. In summary, the oral absorption is characterized by 5 parameters: A_D_, A_R_, A_slow_, a and T (see Table 4).

**Table 4:** Intestinal absorption parameters for oral ramipril: 1) the major ramipril absorption component (gamma function, A, a, and T); 2) a slow, long ramipril component (A_slow_); and 3) direct intestinal conversion and absorption of ramiprilat (gamma function, A_R_, a and T). The total oral dose is 2.5 mg (6010 nanomoles). The total amount absorbed = A + A_slow_+ A_R_.

Subject	Ramipril- Major component			Slow absorp. Amount A_slow _(nmole)	Ramprilat Amount A_R _(nmole)	Total Absorption (nmole)
				
	Amount (A) (nmole)	Time const. (T) (min)	Gamma (a)			
1	2500	90	3	787	200	3487
3	1200	53	9	367	60	1627
4	1600	45	8	432	30	2062
5	1400	48	3.2	0	100	1500
6	1800	35	3	0	100	1900
7	800	25	3	320	41	1161
8	2100	70	2	756	200	3056
9	2200	65	6	0	100	2300
10	1800	60	5.5	0	100	1900
11	1100	35	3	346	100	1546
12	2700	80	4	0	400	3100

Ave (SD)	1745 (597)	55.1 (20.1)	4.4 (2.3)	273 (301)	130 (105)	2149 (755)

All of these features were incorporated into PKQuest and can be activated using a simple interactive menu. The parameters describing the individual PBPK models of each of the two solutes (ramipril and ramiprilat) are entered first. The two solutes are then coupled by defining the parameters V_max _[i,j,m] and K_m _[i,j,m] which are the Michaelis-Menten parameters for metabolism of substrate i to product j in tissue m. It is assumed that the ramipril metabolism is liner, so that Km is set to a very large value and V_max _is set equal to K_m_Cl_int_. An arbitrary number of substrates and products are allowed. Most of the figures used in this paper represent standard PKQuest output.

### Model parameters

The PBPK model is characterized by a large number of parameters. Most of these parameters are fixed and are identical for all subjects (see Table 1). In addition there are 12 parameters that are adjusted (see below) to fit the data for each subject: 4 characterizing the ramiprilat pharmacokinetics (Table 2); 3 characterizing the ramipril metabolism (Table 3); and 5 characterizing the ramipril intestinal absorption (Table 4). A brief description of these parameters is listed here:

**Table 2:** Ramiprilat Adjustable PBPK Parameters: Intrinsic renal clearance, ACE plasma concentration, and liver and kidney cell membrane permeability coefficient. The "average weighted residual error" of the PBPK model for the IV ramiprilat input is listed in the last column.

Subject	Renal Clear. Cl_u _(l/min)	ACE_plasma _(nM)	Membrane Permeability (Ps min^-1^)		IV Ramiprilat Ave. Error
				
			Liver	Kidney	
1	0.55	1.5	0.015	0.0003	0.11
3	0.4	2.35	0.007	0.0015	0.10
4	0.4	1.65	0.018	0.001	0.17
5	0.55	1.13	0.01	0.0006	0.16
6	0.4	1.25	0.015	0.002	0.27
7	0.7	2.25	0.01	0.0006	0.18
8	0.4	2.9	0.01	0.0006	0.11
9	0.37	1.75	0.008	0.0003	0.32
10	0.45	1.45	0.015	0.002	0.13
11	0.45	1.4	0.01	0.0003	0.30
12	0.4	2.25	0.006	0.0003	0.14

Ave (SD)	0.46 (0.10)	1.81 (0.55)	0.011 (0.0039)	0.00086 (0.00067)	0.18 (0.08)

**Table 3:** Ramipril adjustable PBPK parameters: 1) intrinsic liver clearance (Cl_int_L_); 2) intrinsic kidney clearance (Cl_int_K_; 3) the fraction of the liver ramipril clearance that is converted to systemic ramiprilat. The "average weighted residual error'' of the PBPK model plasma ramiprilat following either IV or oral ramipril is listed in the last two columns.

Subject	Cl_int_L _(l/min)	Cl_int_K _(l/min)	Fraction to ramiprilat	IV Ramipril Ave. Error	Oral Ramipril Ave. Error
1	4.8	1.2	0.1	0.2	0.23
3	1	1.5	0.32	0.17	0.25
4	3.6	0.9	0.4	0.23	0.27
5	2	2	0.0	0.17	Poor fit
6	3	1	0.25	0.23	0.28
7	4	1	0.2	0.14	0.14
8	5.5	0	0.4	0.13	0.22
9	3.48	0.52	0.2	0.12	0.38
10	3.5	1.5	0.4	0.30	0.34
11	2.8	1.2	0.3	0.23	0.14
12	6.16	0.84	0.2	0.13	0.2

Ave (SD)	3.62 (1.5)	1.06 (0.53)	0.25 (0.13)	0.19 (0.057)	0.24 (0.077)

#### Ramiprilat ACE binding kinetics

The values determined by Deddish et. al. [21] at 4°C and 300 mM NaCl were used as the default "standard" values for all subjects (see eq. (2)): N site (low affinity): K_N _= .276 nM; k_-N _= 0.0234/min; C site (high affinity): K_C _= 0.039 nM; k_-C _= 0.00168/min.

#### Plasma and tissue ACE concentration

The ACE concentration in normal human plasma determined using a radioimmunoassay varies from 220 to 730 ng/ml [29,52]. (A single genetic polymorphism accounts for 50% of this normal variation). This corresponds to 1.29 to 4.29 nM, assuming a molecular weight of circulating ACE of 170 kDa [53]. The plasma ACE (ACE_plasma_) in the PBPK model is a variable parameter, adjusted to optimize the fit to the experimental data. The model values varied from 1.13 to 2.9 nM for the different subject (Table 2). This is the lower end of the experimental range for humans. The tissue ACE is defined by a standard tissue/plasma ratio (listed in Table 1) and is identical for all subjects. The lung, liver, heart, brain and GI tract ratios are in rough agreement with experimental measurements in rats or rabbits [23,54-56]. The skeletal muscle values are based on needle biopsy measurements in humans [57,58]. Although there are high ACE concentrations in the kidney, most of this activity is in the lumen of the proximal tubule [59], a region that will have limited and slow contact with the circulating ACE inhibitor [56]. The total tissue ACE is 68 times the blood ACE. An essential qualitative feature of the PBPK modeling is the requirement for this large total ACE tissue/blood ratio (see Results). High ACE concentrations have been assigned to the loose connective tissue organs ("tendon" and "other") and to "adipose" tissue based on the observation of Sun et. al. [60] that there was high ACE concentration in subcutaneous connective tissue. Especially important is the high adipose tissue/plasma value of 6.8 because adipose tissue makes a large contribution to the pharmacokinetics because of it large weight (Table 1). Although there is no quantitative adipose date in humans, it has been shown that ACE mRNA is expressed in human adipose tissue [61]. In rats, the subcutaneous fat/plasma ramiprilat binding ratio at 24 hours after an oral dose was about 5 [41].

#### Ramiprilat renal clearance

The intrinsic renal clearance (Cl_u_, eq. (9)) was adjusted to fit the data for each subject (Table 2).

#### Ramiprilat liver and kidney cell membrane permeability and intracellular binding

The product of the two parameters f_u_cell _and Ps determines the rate that the intracellular ramiprilat formed from ramipril enters the systemic circulation (eq. (5)). The value of f_u_cell _for the kidney and liver were assigned arbitrary large values, corresponding to a low binding (Table 1) and the values of Ps were adjusted for each subject (Table 2) to fit the experimental data.

#### Ramipril liver cell membrane permeability and intracellular binding

The ramipril Ps is finite for all tissues, allowing ramipril to distribute in all the body water. The value of f_u_cell _determines the equilibrium volume of distribution and Ps determines the time course of this equilibrium. The fixed values listed in Table 1 were assigned to provide optimal fits to the IV ramipril input.

#### Ramipril metabolic parameters

The intrinsic liver (Cl_int_L_) and kidney (Cl_int_K_) clearance and the fraction of the liver clearance that is converted to ramiprilat (Fr_L_) were adjusted for each subject to fit the oral and IV ramipril data (Table 3).

#### Ramipril intestinal absorption parameters

The values of the 5 parameters describing the intestinal ramipril absorption (A_D_, a, T, A_R_, A_slow_, eqs. (10) and (11)) were adjusted to fit the individual subject data (Table 4).

### ACE assay

It is well recognized that the standard ACE plasma assay in the presence of inhibitors may give a result that differs significantly from the in vivo plasma activity [35,62,63]. The problem arises from the dilution and time dependent effects that become important for the very tight and slow ACE binding. In order to compare the experimental plasma assay measurements with the PBPK model predictions of the fraction of plasma ACE that is complexed with inhibitor, it is necessary to develop a detailed kinetic model of the assay procedure. The following analysis is similar to that of Weisser and Schloos [63], except that they assumed rapid, equilibrium binding, while this analysis use the more general time dependent binding model (eq. (3)).

The ACE assay of the ramipril study used the Vertex kit ACE assay [17] in which a 10 fold dilution of plasma is incubated with 8 mM of the substrate p- [^3^H]benzoylglycylglycylglycine for 60 minutes at 37°C in 100 mM NaCl. Using the same notation as in eq. (2):

I+EC⇄k−CkCIECS+EC⇄KsCSEC→kcatCP+ECSEC=S EC/KsCI+EN⇄k−NkNIENS+EC⇄KsNSEN→kcatNP+ENSEN=S EN/KsNEtC=EC+IEC+SECEtN=EN+IEN+SENIt=I+IEC+IENSt=S+SEN+SEC≈S     (12)
 MathType@MTEF@5@5@+=feaafiart1ev1aaatCvAUfKttLearuWrP9MDH5MBPbIqV92AaeXatLxBI9gBaebbnrfifHhDYfgasaacH8akY=wiFfYdH8Gipec8Eeeu0xXdbba9frFj0=OqFfea0dXdd9vqai=hGuQ8kuc9pgc9s8qqaq=dirpe0xb9q8qiLsFr0=vr0=vr0dc8meaabaqaciaacaGaaeqabaqabeGadaaakeaafaqaaeabdaaaaeaacqWGjbqscqGHRaWkcqWGfbqrdaWgaaWcbaGaem4qameabeaakmaao0aaleaacqWGRbWAdaWgaaadbaGaem4qameabeaaaSqaaiabdUgaRnaaBaaameaacqGHsislcqWGdbWqaeqaaaGccaGLsgIaayjKHaGaemysaK0aaSbaaSqaaiabdweafjabdoeadbqabaaakeaacqWGtbWucqGHRaWkcqWGfbqrdaWgaaWcbaGaem4qameabeaakmaaouaaleaacqWGlbWsdaqhaaadbaGaem4CamhabaGaem4qameaaaWcbeGccaGLsgIaayjKHaGaem4uam1aaSbaaSqaaiabdweafjabdoeadbqabaGcdaGdKaWcbaGaem4AaS2aa0baaWqaaiabdogaJjabdggaHjabdsha0bqaaiabdoeadbaaaSqabOGaayPKHaGaemiuaaLaey4kaSIaemyrau0aaSbaaSqaaiabdoeadbqabaaakeaacqWGtbWudaWgaaWcbaGaemyrauKaem4qameabeaakiabg2da9iabdofatjaaykW7cqWGfbqrdaWgaaWcbaGaem4qameabeaakiabc+caViabdUealnaaDaaaleaacqWGZbWCaeaacqWGdbWqaaaakeaacqWGjbqscqGHRaWkcqWGfbqrdaWgaaWcbaGaemOta4eabeaakmaao0aaleaacqWGRbWAdaWgaaadbaGaemOta4eabeaaaSqaaiabdUgaRnaaBaaameaacqGHsislcqWGobGtaeqaaaGccaGLsgIaayjKHaGaemysaK0aaSbaaSqaaiabdweafjabd6eaobqabaaakeaacqWGtbWucqGHRaWkcqWGfbqrdaWgaaWcbaGaem4qameabeaakmaaouaaleaacqWGlbWsdaqhaaadbaGaem4CamhabaGaemOta4eaaaWcbeGccaGLsgIaayjKHaGaem4uam1aaSbaaSqaaiabdweafjabd6eaobqabaGcdaGdKaWcbaGaem4AaS2aa0baaWqaaiabdogaJjabdggaHjabdsha0bqaaiabd6eaobaaaSqabOGaayPKHaGaemiuaaLaey4kaSIaemyrau0aaSbaaSqaaiabd6eaobqabaaakeaacqWGtbWudaWgaaWcbaGaemyrauKaemOta4eabeaakiabg2da9iabdofatjaaykW7cqWGfbqrdaWgaaWcbaGaemOta4eabeaakiabc+caViabdUealnaaDaaaleaacqWGZbWCaeaacqWGobGtaaaakeaacqWGfbqrcqWG0baDdaWgaaWcbaGaem4qameabeaakiabg2da9iabdweafnaaBaaaleaacqWGdbWqaeqaaOGaey4kaSIaemysaK0aaSbaaSqaaiabdweafjabdoeadbqabaGccqGHRaWkcqWGtbWudaWgaaWcbaGaemyrauKaem4qameabeaaaOqaaiabdweafjabdsha0naaBaaaleaacqWGobGtaeqaaOGaeyypa0Jaemyrau0aaSbaaSqaaiabd6eaobqabaGccqGHRaWkcqWGjbqsdaWgaaWcbaGaemyrauKaemOta4eabeaakiabgUcaRiabdofatnaaBaaaleaacqWGfbqrcqWGobGtaeqaaaGcbaaabaGaemysaKKaemiDaqNaeyypa0JaemysaKKaey4kaSIaemysaK0aaSbaaSqaaiabdweafjabdoeadbqabaGccqGHRaWkcqWGjbqsdaWgaaWcbaGaemyrauKaemOta4eabeaaaOqaaiabdofatjabdsha0jabg2da9iabdofatjabgUcaRiabdofatnaaBaaaleaacqWGfbqrcqWGobGtaeqaaOGaey4kaSIaem4uam1aaSbaaSqaaiabdweafjabdoeadbqabaGccqGHijYUcqWGtbWuaeaaaaGaaCzcaiaaxMaadaqadaqaaiabigdaXiabikdaYaGaayjkaiaawMcaaaaa@E52D@

where E_C_, E_N _and Et_i _are the free C and N site and total enzyme concentration, S is the free concentration of assay substrate and I, I_EC _and I_EN _are the free inhibiter (e.g. ramiprilat) and the inhibitor ACE complex concentration. It is assumed that the substrate concentration (S) is much greater than Et or It and, therefore, S is a constant, equal to the fixed assay concentration. The reported ACE equilibrium dissociation constant (K_s_) for this substrate is 5 mM [17]. This represents the average for the two sites and, since the individual values for each site are not known, it has been assumed that each site has a K_s _of 5 mM.

The true "in vivo" enzyme activity is proportional to the fraction of ACE that is not complexed with inhibitor. This differs from the measured assay activity because the dilution associated with the assay results in a time dependent dissociation of inhibitor during the 60 minute assay. The experimental assay activity (= Assay_60_) is defined as the ratio of the amount of substrate that is hydrolyzed in 60 minutes by the plasma sample in the presence of ACE inhibitor relative to the activity in the same subject when no inhibitor is present (before inhibitor administered). This ratio is the weighted average of the individual catalytic activity of the two sites. In the analysis used here it is assumed that the two sites are independent and what is plotted in the Results is the relative activity of each site which is described by:

Assay60=∫060[EtX−IEX(t)]/(60EtX) dtX=C or N     (13)
MathType@MTEF@5@5@+=feaafiart1ev1aaatCvAUfKttLearuWrP9MDH5MBPbIqV92AaeXatLxBI9gBaebbnrfifHhDYfgasaacH8akY=wiFfYdH8Gipec8Eeeu0xXdbba9frFj0=OqFfea0dXdd9vqai=hGuQ8kuc9pgc9s8qqaq=dirpe0xb9q8qiLsFr0=vr0=vr0dc8meaabaqaciaacaGaaeqabaqabeGadaaakeaafaqabeqacaaabaGaemyqaeKaem4CamNaem4CamNaemyyaeMaemyEaK3aaSbaaSqaaiabiAda2iabicdaWaqabaGccqGH9aqpdaWdXbqaaiabcUfaBjabdweafjabdsha0naaBaaaleaacqWGybawaeqaaOGaeyOeI0IaemysaK0aaSbaaSqaaiabdweafjabdIfaybqabaGccqGGOaakcqWG0baDcqGGPaqkcqGGDbqxcqGGVaWlcqGGOaakcqaI2aGncqaIWaamcqWGfbqrcqWG0baDdaWgaaWcbaGaemiwaGfabeaakiabcMcaPiaaykW7cqWGKbazcqWG0baDaSqaaiabicdaWaqaaiabiAda2iabicdaWaqdcqGHRiI8aaGcbaGaemiwaGLaeyypa0Jaem4qamKaaGPaVlabd+gaVjabdkhaYjaaykW7cqWGobGtaaGaaCzcaiaaxMaadaqadaqaaiabigdaXiabiodaZaGaayjkaiaawMcaaaaa@65C3@

The concentration of the inhibitor-enzyme complex (I_EX_(t)) decreases during the 60 minute incubation as inhibitor dissociates because of dilution and competition with substrate. The value of I_EX_(t)) is determined from a solution of a differential equation obtained from eq. (12). (See the additional file 1 ACE_supplemental_31oct05.doc, section II for details).

### Experimental data

The PBPK model was fitted to the results described previously by van Griensven et. al. [38] of a 3 way crossover trial using 12 healthy young (19 to 28 years), non-obese (average weight = 76.25 kg and height = 1.86, BMI 19.9 to 24) males [38]. Only 11 subjects were used in the analysis because there was no oral data for subject 2. The three arms of the study were: 1) 2.5 mg oral ramipril; 2) approximately 2.5 mg (range 2.59 – 2.97) IV ramipril given as a constant 1 min infusion; and 3) approximately 2.5 mg (range 2.59 – 3.19) IV ramiprilat given as a 1 min infusion.

The value of the antecubital vein plasma ramipril and ramiprilat and the serum ACE activity (Ventrix kit) was determined at 0, 5, 10, 20, 30, 45, 60, 90 minutes and 2, 3, 4, 5, 6, 8, 10, 12, 24, and 72 hours. It is assumed that the antecubital vein concentration can be approximated by the central vein concentration. The urine concentration of ramipril, ramiprilat and the major metabolites was determined at varying times out to 72 hours. The plasma ramiprilat was determined by a ^125^I-radioimmunoassay using rabbit antibodies to ramiprilat. The plasma ramipril was determined by measuring the difference between the ramiprilat concentration before and after enzymatic hydrolysis of the ramipril to ramiprilat. This procedure could lead to relatively large errors in the ramipril measurements in the presence of high ramiprilat concentrations and is, presumably, the explanation for the large fluctuations in the plasma ramipril concentrations at long times (see below). The reported detection limits were 1 ng/ml (2.4 nM) for ramipril and 0.5 ng/ml (1.3 nM) for ramiprilat [64].

The selection of the parameters was based entirely on simple trial and error and subjective adjusting of parameters. The current version of PKQuest does not allow automated fitting when more then one data set is used. (In this application, parameters must be optimized to fit 4 different data sets: IV ramiprilat, IV ramipril, oral ramipril and urine ramiprilat.) In addition, the fitting is complicated by choices of how to weight the data. Because of the large fluctuations in plasma ramipril, less weight was given to the model predictions at low ramipril concentration. The individual model predictions for each subject and each data set is shown in the Results section. This is the best qualitative measure of the model. In addition, the "average weighted residual error" is tabulated for each individual data set (Tables 2, 3, 4):

WRE=(1/N)∑i=1N|Experimentali−Modeli|/Modeli     (14)
 MathType@MTEF@5@5@+=feaafiart1ev1aaatCvAUfKttLearuWrP9MDH5MBPbIqV92AaeXatLxBI9gBaebbnrfifHhDYfgasaacH8akY=wiFfYdH8Gipec8Eeeu0xXdbba9frFj0=OqFfea0dXdd9vqai=hGuQ8kuc9pgc9s8qqaq=dirpe0xb9q8qiLsFr0=vr0=vr0dc8meaabaqaciaacaGaaeqabaqabeGadaaakeaacqWGxbWvcqWGsbGucqWGfbqrcqGH9aqpcqGGOaakcqaIXaqmcqGGVaWlcqWGobGtcqGGPaqkdaaeWbqaamaaemaabaGaemyrauKaemiEaGNaemiCaaNaemyzauMaemOCaiNaemyAaKMaemyBa0MaemyzauMaemOBa4MaemiDaqNaemyyaeMaemiBaW2aaSbaaSqaaiabdMgaPbqabaGccqGHsislcqWGnbqtcqWGVbWBcqWGKbazcqWGLbqzcqWGSbaBdaWgaaWcbaGaemyAaKgabeaaaOGaay5bSlaawIa7aiabc+caViabd2eanjabd+gaVjabdsgaKjabdwgaLjabdYgaSnaaBaaaleaacqWGPbqAaeqaaOGaaCzcaiaaxMaadaqadaqaaiabigdaXiabisda0aGaayjkaiaawMcaaaWcbaGaemyAaKMaeyypa0JaeGymaedabaGaemOta4eaniabggHiLdaaaa@684F@

Given the number of adjustable parameters and the complexity of the experimental data, this subjective aspect of the model fitting cannot be avoided. Several subjects had a bimodal ramiprilat concentration curve following IV ramipril with a sharp peak at 5 or 10 minutes that is not predicted by the model and may be a result of hydrolysis occurring in the hand and arm tissues drained by the antecubital vein. These points were not included in the calculated weighted error. As demonstrated by the parameter variation studies described in the Results, some parameters are highly constrained, while others have wide ranges of possible values. The major emphasis of this analysis is on the potential of the PBPK approach to determine the time and dosage dependence of the ACE inhibition, not on the quantitative value of the parameters.

## Results

### IV ramiprilat infusion

Of the 3 sets of data that were modeled, the IV ramiprilat is the simplest because the ramiprilat systemic input is known and there is no ramipril present. The ramiprilat PBPK parameters are: 1) the 4 ACE binding constants (K_N_, K_C_, k_-N_, k_-C_, eq. (2)); 2) the renal intrinsic clearance (Cl_u_, eq. (9)) and 3) the total ACE concentration in plasma and each tissue (Et^i^, 1 = 1..12). (The ACE albumin binding (eq. (4)) was determined directly from experimental measurements). Only two of these parameters (Cl_u _and Et^plasma^) are varied to optimize the fit for each subject. The other parameters were fixed at their "standard values" (see Methods). Other PBPK parameters, such as tissue volume and blood flow, extracellular water, tissue albumin, etc. are taken from the default "standard human" data set that was determined previously by application of PKQuest to other solutes.

The confidence limits of values of these model parameters were determined by an analysis of the sensitivity of the model predictions to variations in the parameters. A summary of this analysis is described here. (See the additional file 1 ACE_supplemental_31oct05.doc section III for a detailed sensitivity analysis). The values of the renal clearance (Cl_u_), the plasma ACE and the total tissue ACE are uniquely determined (to within about ± 30%) by the experimental data. As Brockmeier [35,36] emphasized, the renal clearance of ramiprilat provides a direct measurement of the fraction that is free in plasma and the experimental urine ramiprilat excretion data has a crucial role in the assignment of a unique set of parameters. The model results are relatively insensitive to the distribution of the total ACE among the different tissues. The values of the ramiprilat binding constants (K_N_, K_C_, k_-N_, k_-C_) cannot be uniquely determined by the experimental data and only qualitative constraints can be placed on them. Values of k_-N _and k_-C _varying from 10% of the standard value, up to infinity provide similar fits. Only the average value of K_N _and K_C _can be uniquely assigned. For example, an identical K_N _= K_C _= 0.140 nM for both sites (equivalent to the single binding site assumption of Brockmeier [35,36]) provides as good a fit to the data as the "standard" model (K_N _= .276, K_C _= 0.039 nM).

**Figure 3:**
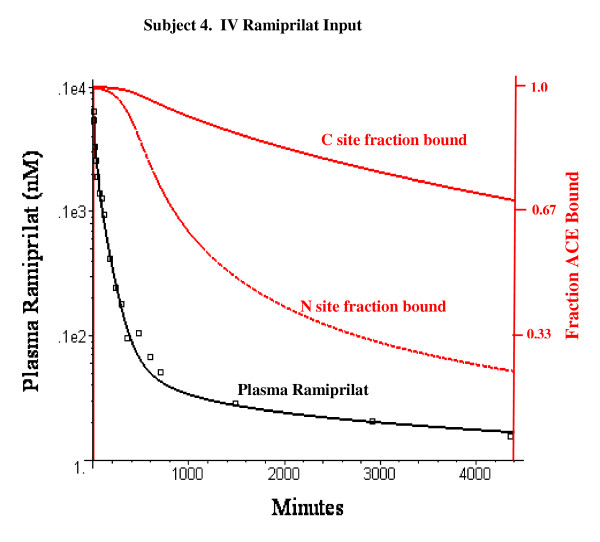
Semi-log plot of model plasma ramiprilat (nanomoles/liter) following IV ramiprilat in subject 4 (black line) and the corresponding fraction of the C and N site of plasma ACE that is occupied by ramiprilat (red lines). The open squares are the experimental plasma ramiprilat values.

Figure 3 shows the relationship between the plasma concentration (black) and the fraction of the N and C sites that are occupied by ramiprilat (red) for subject 4. The plasma ACE for subject 4 is 1.65 nM, so that the total binding site concentration is 3.3 nM. At early times (< 400 minutes), most of the ramiprilat is free, both sites are nearly 100% occupied, and the plasma ACE is determined by the renal clearance (Cl_u_) and is independent of the ACE concentration and binding kinetics. At long times most of the ramiprilat is bound and the plasma concentration is determined primarily by the ACE concentration and binding kinetics. At 72 hours, the total plasma ACE equals 1.68 nM, the C site is 70% occupied, the N site is 24% occupied and 95% of the ramiprilat is bound to ACE.

**Figure 4:**
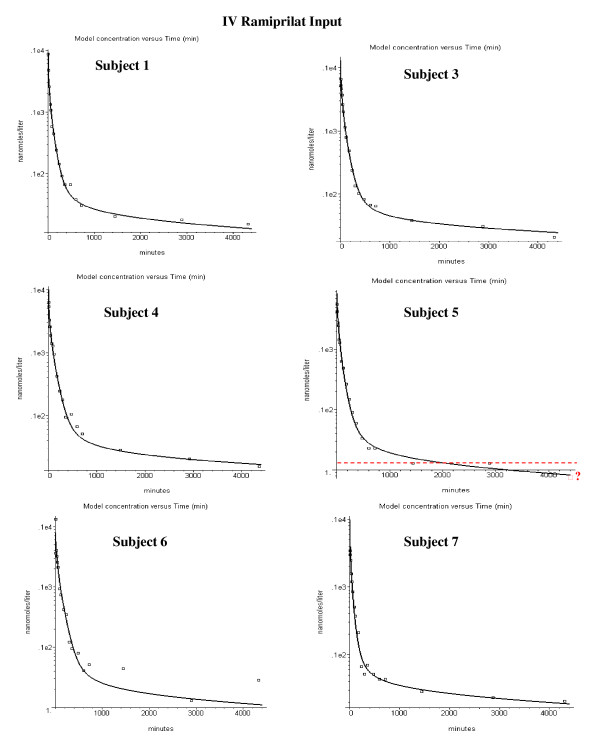
Plasma ramiprilat concentration following IV ramiprilat for subjects 1 to 7.

**Figure 5:**
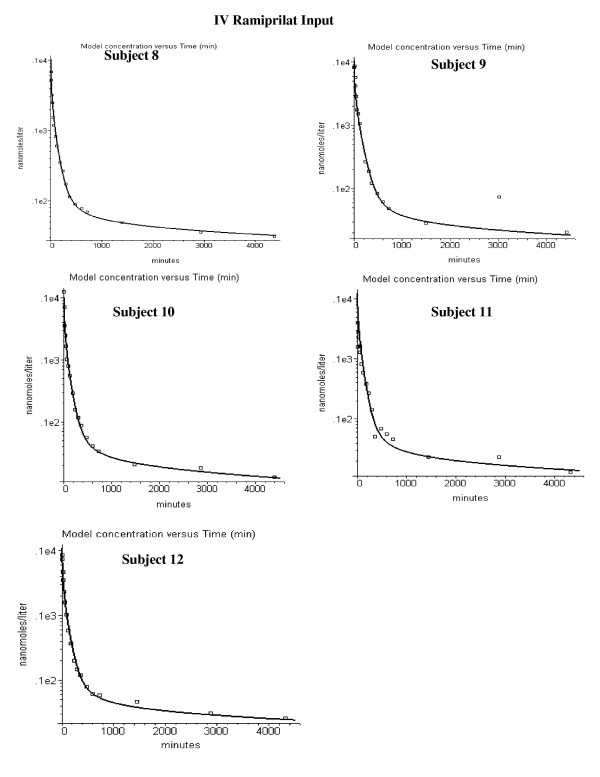
Plasma ramiprilat concentration following IV ramiprilat for subjects 8 to 12.

Figures 4 and 5 show the model fits for the IV ramiprilat input data for each of the 11 subjects. Two parameters were adjusted for each subject – the renal clearance (Cl_u_) and the plasma ACE concentration. The values of these two parameters and the weighted average error (eq. (14)) are listed in Table 2. The ramiprilat binding constants and the ACE tissue/plasma ratio are identical for all subjects. The agreement between the PBPK model predictions and the experimental data is quite good, with an average error of 18%.

### IV and oral ramipril infusion

**Figure 6:**
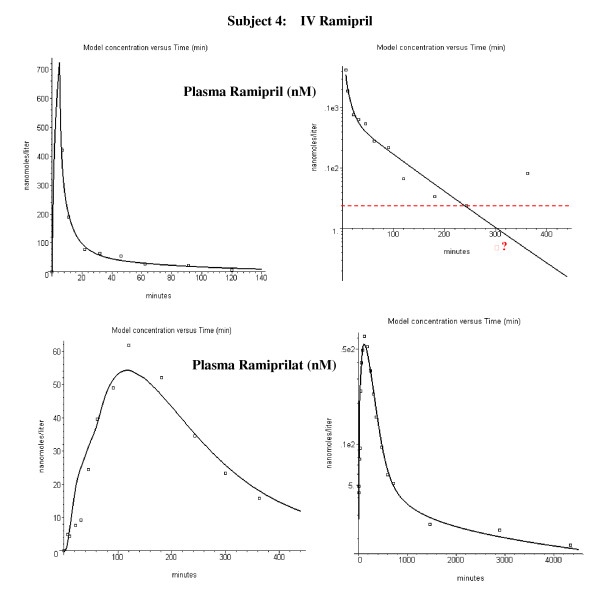
Plasma ramipril (top panels) and ramiprilat (bottom panels) following IV ramipril for subject 4. Left column: early time data on absolute scale. Right column: long time data on semi-log scale. The open squares are the experimental plasma values. The dashed red line indicates ramipril detectable limit and the red square indicates that the plasma value was below this limit.

**Figure 7:**
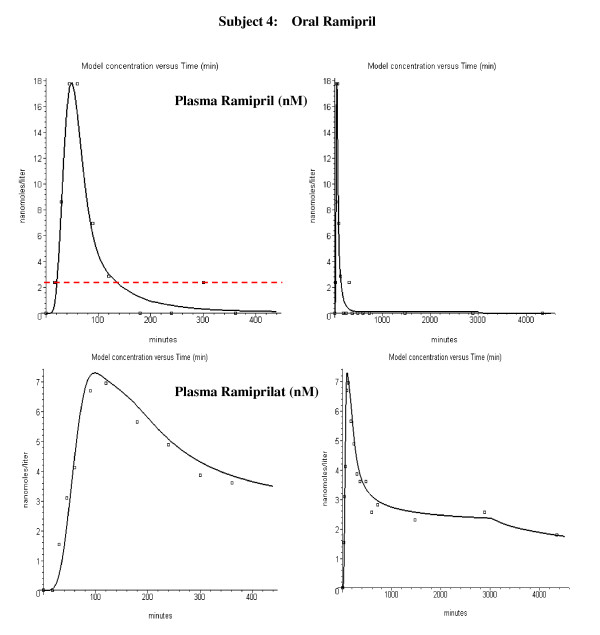
Plasma ramipril (top panels) and ramiprilat (bottom panels) following oral ramipril for subject 4. Left column: early time data. Right column: all data. The open squares are the experimental plasma values. The dashed red line indicates the analytical detection limit for ramipril.

Figure 6 shows the plasma ramipril and ramiprilat data following an IV bolus infusion of 2.89 mg ramipril in subject 4. The lines in fig. 6 are the PBPK model predictions using the optimized standard parameters in Tables 1 and 2. The ramipril is rapidly removed from the systemic circulation while the ramiprilat appearance is significantly delayed. This delay results from the intracellular accumulation and slow release of the ramiprilat produced from ramipril in the liver and kidney (fig. 1). There are large fluctuations in the plasma ramipril curve at long time and low concentrations that cannot be attributed to enterohepatic recirculation since it would require impossibly large and rapid rates of secretion and reabsorption. It is assumed that this fluctuation is an artifact of the analytical ramipril methodology discussed in the Methods. Seven parameters are required to model the IV ramipril experiments (see Methods): Cl_int_L_, Cl_int_K _and Fr_L _describe the liver and kidney metabolism of ramipril and its conversion to ramiprilat; Ps_L _and Ps_K _describe the rate that this ramiprilat leaves the liver and kidney and enters the systemic circulation; Ps_T _describes the rate that ramipril enters the intracellular water of the peripheral tissues and f_u_cell _describes the intracellular fraction unbound which determines the ramipril equilibrium volume of distribution. Figure 7 shows the plasma ramipril and ramiprilat following an oral dose of 2.5 mg ramipril in the same subject. Five additional parameters (A_D_, a, T, A_R_, A_slow_) are required to model the oral ramipril intestinal absorption.

In total, 12 new parameters are required to describe the oral and IV ramipril data. Although it is not possible to specify a unique, quantitative parameter set, an analysis of the sensitivity of the model fits to variation in the parameters indicates that all of these parameters make significant contributions to the quality of the model fit. This sensitivity analysis is summarized here. (See the additional file 1 ACE_supplemental_31oct05.doc section IV for a detailed analysis).

The 3 metabolic parameters (Cl_int_L_, Cl_int_K _Fr_L_) are required to describe conversion of ramipril to systemic ramiprilat in the kidney and liver. The requirement for these two organ components arises from the different way the liver and kidney act on an IV (no first pass metabolism) versus an oral input (with first pass metabolism). A significantly poorer fit is obtained if only liver metabolism is included in the model. The parameters Ps_L _and Ps_K _characterize the rate that the intracellular ramiprilat produced from ramipril enters the systemic circulation from the liver and kidney. They are adjusted to fit the delayed systemic appearance of ramiprilat following IV or oral ramipril and are determined within factors of about 30% by the experimental data. The sensitivity analysis indicates that a finite peripheral tissue cell membrane ramipril permeability (Ps_T_), which allows the ramipril to distribute in all the body water, clearly provides a better fit then restricting the ramipril to the extracellular space. Any permeability value ranging from the standard value up to infinity provides an equally good fit.

Five parameters are required to describe the intestinal absorption of oral ramipril. The major component is described by the 3 parameter (A, a and T) gamma distribution function (eq. (10)) which is routinely used to describe oral uptake in PKQuest [5]. In some subjects there is an early peak in the plasma ramiprilat following oral ramipril that can only be explained by adding an absorption component (A_R_) where the ramipril is directly converted to ramiprilat either in the intestinal lumen or epithelial cell and directly absorbed, bypassing the slower liver and kidney pathways. Finally, in some subjects the plasma ramiprilat following oral ramipril remained constant between 200 and 3000 minutes (about 2 days), requiring the addition of a slow delayed ramipril absorption component (A_slow_), presumably representing enterohepatic recirculation and/or large intestinal absorption.

**Figure 8:**
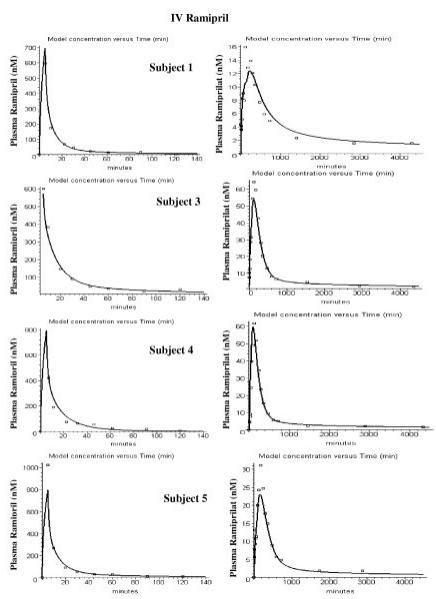
PBPK model (solid line) plasma ramipril (left column) and ramiprilat (right column) following IV ramipril for subjects 1 to 5. The open squares are the experimental data.

**Figure 9:**
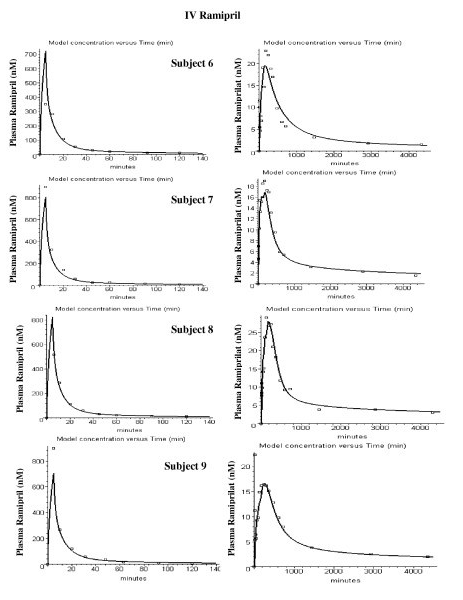
PBPK model (solid line) plasma ramipril (left column) and ramiprilat (right column) following IV ramipril for subjects 6 to 9. The open squares are the experimental data.

**Figure 10:**
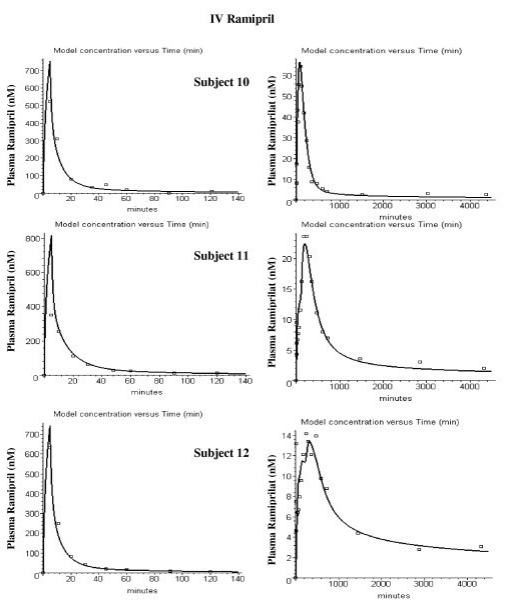
PBPK model (solid line) plasma ramipril (left column) and ramiprilat (right column) following IV ramipril for subjects 10 to 12. The open squares are the experimental data.

**Figure 11:**
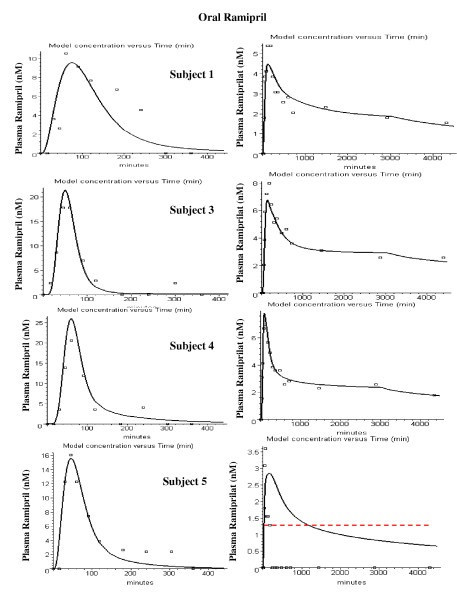
PBPK model (solid line) plasma ramipril (left column) and ramiprilat (right column) following oral ramipril for subjects 1 to 5. The open squares are the experimental data.

**Figure 12:**
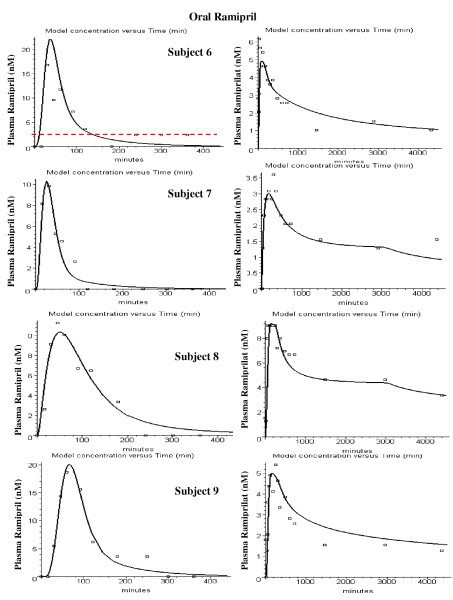
PBPK model (solid line) plasma ramipril (left column) and ramiprilat (right column) following oral ramipril for subjects 6 to 9. The open squares are the experimental data.

**Figure 13:**
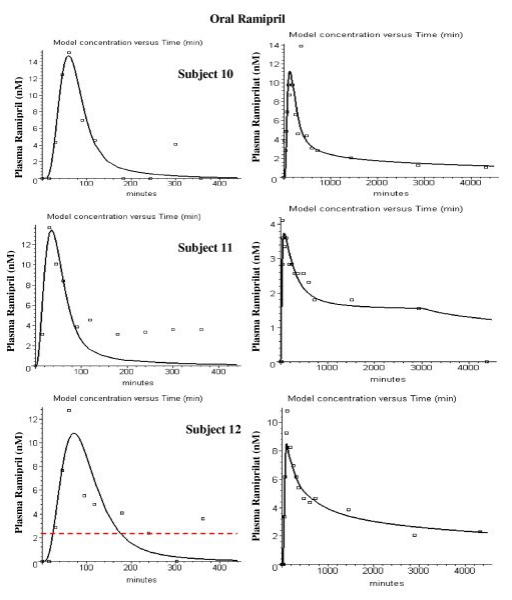
PBPK model (solid line) plasma ramipril (left column) and ramiprilat (right column) following oral ramipril for subjects 10 to 12. The open squares are the experimental data.

**Figure 14:**
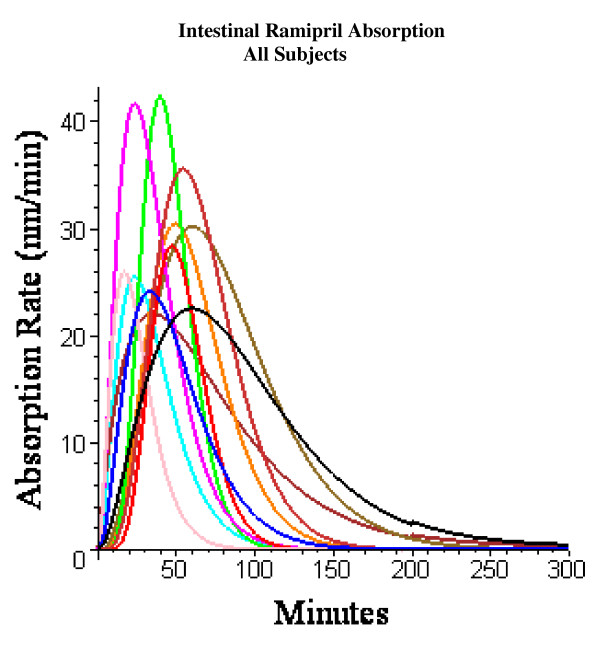
PBPK model (solid line) rate of intestinal ramipril absorption (sum of fast and slow components) for all subjects.

Figures 8, 9, 10, 11, 12, 13 show the model plots and Tables 2, 3 and 4 list the values of the adjustable parameters for the IV and oral ramipril input for each of the 11 subjects. The plots provide a good view of the large individual variation. For example, although the plasma ramipril concentration following an IV ramipril dose is nearly identical for subjects 1 and 4 (fig. 8), the resulting peak plasma ramiprilat concentration for subject 4 is nearly 5 times higher than for subject 1 (fig. 8). This is an indication of the large subject to subject variation in ramipril metabolism. It can be seen that the PBPK model provides an adequate description of this variation, with an average weighted error of about 20% (Tables 2 and 3). The only exception is subject 5 (fig. 11) where the plasma ramiprilat following oral ramipril falls much more rapidly to non-detectable levels than the model predicts. Figure 14 shows a plot of the sum of the intestinal absorption rate of the major and slow ramipril components for all subjects. (This does not include the direct ramiprilat absorption component.) The total oral absorption varies from 1500 to 3487 nanomoles, representing 25 to 58% of the 2.5 mg dose. The average total absorption was 35% of the oral dose. This is 12% greater than the 23.2% total amount of ramipril and its metabolites that was recovered in the urine in these same subject [38] and is consistent with measurements of Verho et. al. [65] that an additional 17% of the oral dose is excreted in the bile in humans.

### ACE assay

In addition to the ramipril and ramiprilat concentrations, the experimental ACE activity was also determined for each plasma sample. What is reported is the fractional enzyme activity which is defined as equal to the amount of the test substrate hydrolyzed in 60 minutes relative to the hydrolysis in the absence of ACE inhibitor (see Methods). This activity is proportional to the fraction of the ACE sites that are unoccupied by ramiprilat. The assay activity is not equal to the true in vivo activity because the ramiprilat dissociates (and enzyme activity increases) during the 60 minute assay because of assay dilution and competition with the test substrate.

**Figure 15:**
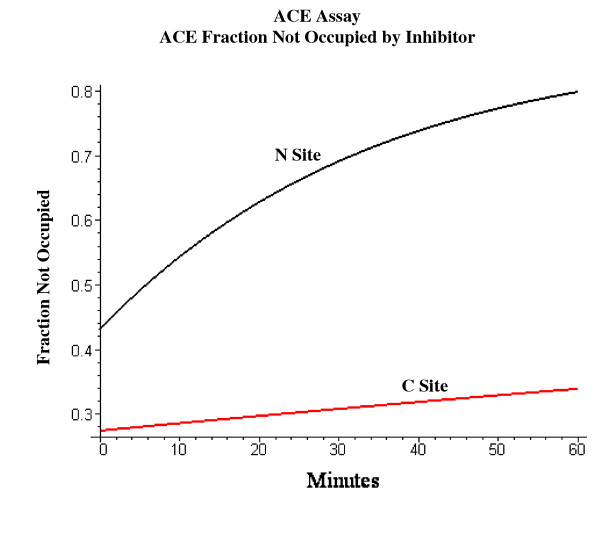
Variation of the ACE activity (= fraction of N and C ACE sites not occupied by ramiprilat) during the 60 minute incubation with the test substrate during the standard ACE assay. The activity at time 0 represents the true in vivo fractional ACE activity.

**Figure 16:**
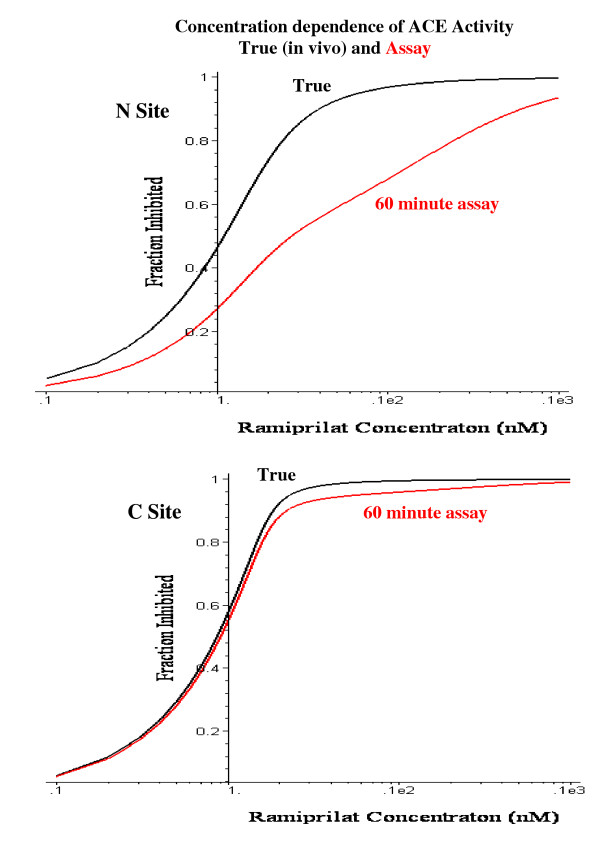
Comparison of true in vivo fraction of C and N site of ACE that is inhibited by ramiprilat (black) versus the activity determined from the standard ACE assay (red) as a function of the plasma ramiprilat concentration.

Figure 15 shows an example of the change in activity (as a fraction of the maximum activity when no inhibitor is present) during the 60 minute ACE assay. The ramiprilat concentration (before dilution) for this plot is 1.3 nM, 79% of the concentration of each ACE site for subject 4 (1.65 nM). It can be seen that for the high affinity C site (K_C _= 0.039 nM) there is little change in inhibitor binding, while for the lower affinity N site (K_N _= .276 nM), there is a large change during the 60 minutes as the ramiprilat dissociates because of the assay dilution. The activity at time = 0, before any ramiprilat dissociation occurs, corresponds to the "true", in vivo plasma activity. The measured activity is the average activity during the 60 minutes incubation (Assay_60_, eq. (13)). Figure 16 shows a comparison of the "true" in vivo fractional ACE inhibition (at t = 0) versus the 60 minute assay activity for varying concentrations of the plasma ramiprilat. For the high affinity C site, the experimental assay does not differ significantly from the true activity, while for the lower affinity N site the assay activity can be as much as 70% greater than the true activity.

**Figure 17:**
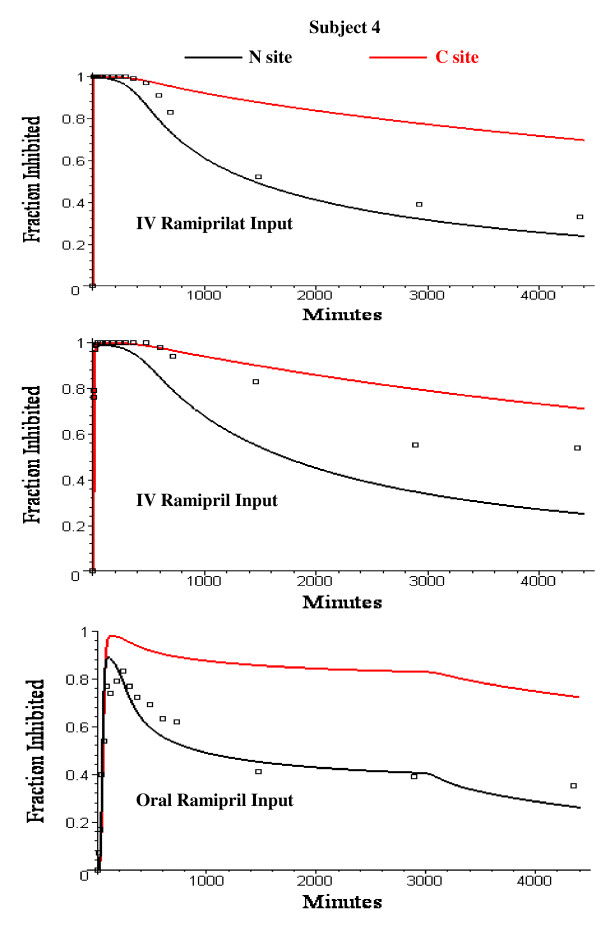
PBPK model prediction of the true in vivo fraction of C (red) and N (black) site of plasma ACE that is inhibited by ramiprilat (black) following IV ramiprilat (top), IV ramipril (middle) and oral ramipril (bottom) for subject 4. The open squares are the experimental plasma ACE activity determined by the standard assay for subject 4.

The plasma ACE activity as a function of time was determined experimentally for all 3 arms of the study using the 60 minute Vertex assay. Figure 17 shows a comparison of the PBPK model predictions of the "true" in vivo fraction of the C and N sites that are inhibited versus the experimentally measured ACE fraction inhibited (squares) for the IV ramiprilat, IV ramipril and oral ramipril input in subject 4. If the two sites are independent, the experimental ACE activity should be the weighted average of the individual catalytic activity of the N and C sites which have different substrate selectivity [19,21,66]. Since there is no information about the individual site activity for the substrate used in this assay ([^3^H]benzoylglycylglycylglycine), one cannot determine which one (or both) of the sites is being assayed by this procedure. The ACE activity is closest to that of the N site suggesting that this substrate is hydrolyzed primarily by the N site. (For the N site, the assay activity should be about 50% greater than the "true" fraction inhibited, see fig. 16). The relationship between the fraction of the sites occupied by ramiprilat and the experimental activity will be further complicated if there is negative cooperativity between the sites, as suggested by Skirgello et. al. [20]

## Discussion

### Validity of the PBPK model

Although the PBPK model (fig. 1) is complicated, as discussed above (Results) it represents the minimum arrangement required to describe the experimental ramipril/ramiprilat kinetics. There is direct experimental evidence that qualitatively supports all of the model assumptions. The following lists the major assumptions of the model along with the experimental support.

1. Plasma ACE has two independent high affinity binding sites (N and C terminal) with kinetics described by eq. (2). This is directly supported by in vitro measurements [19,21]. However, the only known values of the kinetic constants for ramiprilat binding are at 4°C and 300 mM NaCl (see Methods). For lack of better information, these are the values that have been used in the PBPK model.

2. Tissue ACE is identical to circulating plasma ACE. The strongest support for this assumption is that it has been shown that plasma ACE is directly derived from the membrane bound tissue ACE by post-translational proteolytic cleavage [28,29]. This is a critical assumption. Tissue ACE represents more than 90% of the total ACE and is the site of the clinical action of the ACE inhibitors [9]. This assumption allows one to use a single set of binding constants derived from in vitro measurements on tissue ACE to predict the plasma and tissue kinetics.

3. Ramiprilat is an extracellular solute and ramipril distributes in all the body water. The ramiprilat assumption is supported by the relatively low ramiprilat octanol/water distribution coefficient of 0.011 at pH 7.0 [42]. Direct support for this assumption is provided by the observation that ramiprilat has a very slow rate of crossing the blood-brain barrier, with a free ramiprilat CSF/Plasma ratio of about 0.02 2 hours after an IV ramiprilat injection in dogs [67]. This assumption simplifies the description of the ramiprilat distribution kinetics since it allows the use of the previously determined PBPK set of extracellular volume and albumin binding parameters [6]. (The special transport systems that extrude ramiprilat from liver and kidney cells are an exception to this assumption). In contrast, the free ramipril CSF/Plasma ratio 2 hours after IV ramipril is about 2 [67], supporting the model assumption that ramipril has a high cell membrane permeability and distributes in all the body water.

4. Both the liver and kidney cells take up ramipril, hydrolyze it to ramiprilat and release it back to the circulation. The PBPK modeling of the rate of uptake, metabolism and release determines the kinetics of plasma ramipril and ramiprilat following a ramipril input. The investigations of Pang and colleagues [43-49] of enalapril and enalaprilat kinetics in perfused rat liver and kidney provide direct qualitative confirmation of this assumption. For the subjects in this study, 0 to 60% (mean = 25%) of the total ramipril metabolism occurs in the kidney (Table 3). This is consistent with the observation that ramipril clearance decreases by about 50% in patients with renal failure [68-70]. (One publication found no change in ramipril clearance in renal failure [71].)

5. The only site of ramiprilat removal is renal clearance. This is consistent with the observation of a 3 to 4 fold decrease in ramiprilat clearance in renal failure patients with a 3 to 4 fold reduction in creatinine clearance [69,71].

6. All of the ramiprilat produced from ramipril by the kidney is released back to the systemic circulation and is not excreted in the urine. This can be directly tested by comparing the renal ramiprilat clearance for the IV ramipril input versus the IV ramiprilat input. If some of the ramipril is directly converted to ramiprilat and excreted by the kidney then one would expect a greater apparent ramiprilat clearance with the IV ramipril input. Comparing the average value of this clearance in the 11 subjects for the first 240 minutes when most of the ramiprilat is unbound, there was no significant difference in the clearances for the two inputs (0.364 vs 0.365 l/min).

7. There are 3 components of intestinal absorption of oral ramipril (Table 4): 1) the major ramipril component described by a gamma function (eq. (10)) that is similar to what has been used to describe the absorption of many other drugs [5] (81% of the total absorption); 2) a much slower ramipril component that extends out to 3000 minutes (13%); 3) direct intestinal conversion of ramipril to ramiprilat which is then absorbed (6%). The slow component could represent either enterohepatic recirculation [65] or slow absorption from the large intestine. Although there is no evidence in humans to support the existence of the direct ramiprilat absorption, Pang et. al. [48] have observed this component using a perfused rat intestine preparation.

There are a total of 12 adjustable parameters in the PBPK model: 4 for ramiprilat (renal clearance, plasma ACE, and renal and liver cell membrane permeability, Table 2); 3 describing the ramipril metabolism and conversion to ramiprilat (Table 3); and 5 characterizing the rate of oral ramipril absorption (Table 4). Although this may seem like a lot, it should be emphasized that they are used to describe 3 separate data sets (IV ramiprilat, IV ramipril and oral ramipril) along with the renal ramiprilat excretion data. In addition to describing the distribution and clearance of ramipril and ramiprilat, this PBPK model also describes: 1) the liver and kidney cellular uptake and metabolism of ramipril; 2) the fraction of the total ramipril metabolism that is converted to ramiprilat; 3) the rate of intracellular ramiprilat production in the liver and kidney; 4) the rate that intracellular ramiprilat enters the systemic circulation; 5) the intestinal absorption of ramipril; and 6) the time dependent binding to plasma and tissue ACE. For comparison, a simple linear compartmental model would require a minimum of 15 parameters: 2-compartment model description of the pharmacokinetics of the individual ramipril (4 parameters) and ramiprilat (4 parameters); conversion of ramipril to ramiprilat (2 parameters) and intestinal absorption (5 parameters). Additional parameters would be required to describe the non-linearity and the individual C and N binding sites. The PBPK model uses experimentally realistic parameters and, as shown in figs. 4 and 5 and figs. 8, 9, 10, 11, 12, 13, provides a good description of the individual subject variation, with an average weighted residual error of about 20% for all the data (Table 2 and 3).

### Clinical implications

There is now a general consensus that the most rational approach to answering questions about dosage and efficacy of the different ACE inhibitors should be based on an analysis of the quantitative inhibition of tissue ACE [9]. The most direct approach to this question is to measure the plasma ACE activity as a function of time. This should provide a good measure of tissue ACE inhibition because, as is shown below, tissue and plasma ACE should have the same fractional inhibition at long times. Unfortunately, the standard plasma ACE activity measurements have an uncertain relationship to the true in vivo activity for two reasons: 1) During the assay, the ACE activity decreases because of dilution of the ACE inhibitor (see figs. 15 and 16); and 2) the assay represents a weighted average of the sum of the activity of the N and C sites for the test substrate used in the assay (see fig. 17). The activity of the C site is most important for the renin-angiotensin system, since, at physiological conditions (37°C, 50 mM NaCl) the C site has a 3 fold higher k_cat _and a 2.7 fold higher k_cat_/ K_m _for hydrolysis of angiotensin I than the N site [66]. The C site also has a higher activity for bradykinin and substance P hydrolysis [72]. Since the physiological inhibitor and substrate binding constants and the specific assay substrate activity of the two sites are not known, it is not possible to relate the assay activity to the true in vivo activity. Another approach that has been used in animals is direct measurements of tissue ACE activity using radioinhibitor binding [26,59]. However, these displacement binding measurements suffer from the same problems of dilution and competition as the plasma assay measurements.

The major advantage of a PBPK model is that it has the potential to predict the detailed time dependent tissue ACE activity for arbitrary dosage regimens of the different ACE inhibitors. The accuracy of these predictions depends, of course, on the accuracy of the PBPK model. The major uncertainty in the model described here is the uncertainty in the value of the ramiprilat binding constants at physiological conditions. Although the values assumed for the "standard model" which are based on measurements at 4°C and 300 mM NaCl provide good fits to the data, other combinations of binding constants for the two sites also provide good fits. For example, an equally good fit is obtained by assuming that the two sites are identical and have a K of 0.14 nM, intermediate between the standard value for the C site (K_C _= 0.039 nM) and the N site (K_N _= .276 nM). The following analysis uses the standard model fixed parameters and the average values of the adjustable parameters (Table 2, 3, 4) to illustrate the potential of this PBPK analysis. It is assumed that there is no slow oral absorption component (5 of the 11 subjects had no slow component, Table 4).

**Figure 18:**
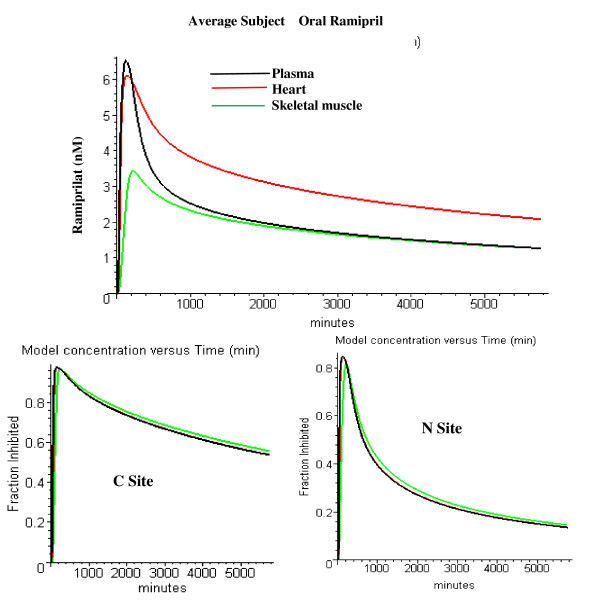
Top: model plasma (black), heart (red) and skeletal muscle (green) ramiprilat concentration following oral ramipril in a subject with average PBPK parameters. Bottom: fraction of C site (left) and N site (right) ACE in plasma (black), heart (red) and skeletal muscle (green) that is inhibited by ramiprilat following oral ramipril in same average subject.

The top panel in fig. 18 shows the PBPK plasma ramiprilat concentration for a 4 day period following a single 2.5 mg oral dose of ramipril. Also plotted are the ramiprilat concentration in a high (heart) and low (skeletal muscle) blood flow tissue. After about 600 minutes, most of the ramiprilat is bound to ACE and the concentration in the different tissues primarily reflects the difference in the tissue ACE concentration (ACE tissue/plasma = 1.0 for muscle and 1.7 for heart, Table 1). The bottom two panels in fig. 18 show the fraction of N and C ACE sites that are occupied by ramiprilat (i.e. the fraction inhibited) in the plasma and the two tissues. The ACE in the high blood flow heart rapidly equilibrates with the plasma, while the low flow skeletal muscle lags slightly behind the plasma. The differences in fraction inhibited in the different tissues are small and, as a first approximation, the ACE fractional activity in plasma is an accurate measure of the fractional activity in tissue.

For this 2.5 mg oral dose, which is the lowest that is clinically recommended, the higher affinity C site is 80% inhibited after 1 day and 55% inhibited after 4 days. Since the C site has the primary action on the renin-angiotensin system (see above), a single, daily 2.5 mg dose of ramipril should reduce the average activity of this system by about 80% or more (assuming the "standard" binding constants and independent sites).

**Figure 19:**
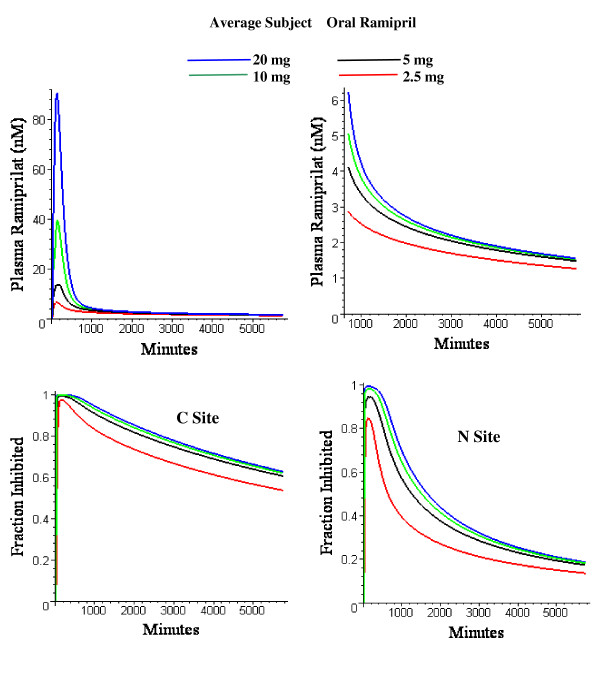
Top: model plasma ramiprilat for entire experimental period (left) and at long times (right) following 2.5 mg (red), 5 mg(black), 10 mg (green) or 20 mg (blue) oral ramiprilat in "average" subject. Bottom: Fraction of C site (left) or N site (right) inhibited in same subject for same set of oral ramipril doses.

Figure 19 shows the plasma ramiprilat and the fractional inhibition of the C and N site as the oral ramipril dose varies from 2.5 to 20 mg, the clinically recommended range. This figure dramatically illustrates the non-linearity in the pharmacokinetics produced by the high ACE affinity binding. At short times, the plasma ramiprilat is roughly proportional to the dose and most of it is unbound and is rapidly cleared by the kidney. At long times, most of the ramiprilat is bound to ACE and the plasma concentration is determined primarily by the plasma ACE concentration and is relatively independent of the dose. The fractional ACE inhibition is also non-linear. This analysis suggests that increasing the oral dose above 5 mg/day should not significantly increase the clinical action of the drug since it does not produce a significant increase in the fraction of ACE that is inhibited.

**Figure 20:**
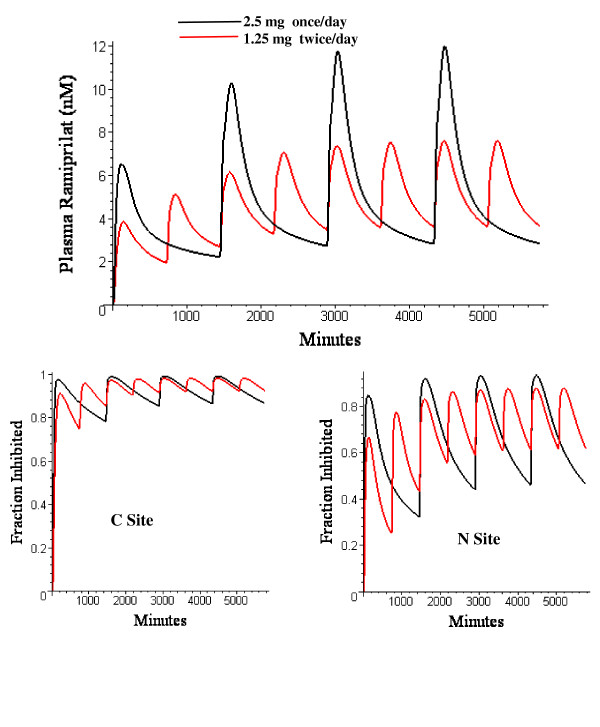
Top: five day plasma ramiprilat for either once per day 2.5 mg oral ramipril (black) or twice per day 1.25 mg oral ramipril (red). Bottom: Fraction of C site (left) or N site (right) inhibited in same subject for same multiple dose regimen.

Figure 20 shows the plasma ramiprilat and the fractional inhibition of the C and N site for multiple oral ramipril doses of either 2.5 mg once per day or 1.25 mg twice a day. The once/day results agree with the experimental observation that the plasma concentration reaches a steady state within one week with increases in peak concentration of about 1.5 times that of the first dose [73]. An interesting prediction of this analysis that may be clinically relevant is that for the lower affinity N site the twice/day regimen results in a significant increase in the average inhibition.

**Figure 21:**
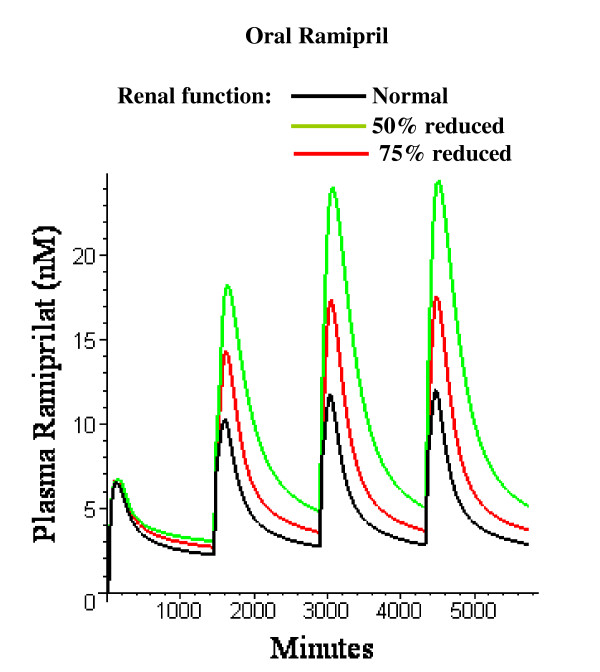
Influence of reductions in renal function on plasma ramiprilat following 4 days of 2.5 mg oral ramipril once per day in the "average" subject.

Figure 21 shows the effect of a reduction in renal function on the plasma ramiprilat for an oral ramipril dose of 2.5 mg once per day for 4 days. Renal function has two effects on the pharmacokinetics. About 23% of the conversion of ramiprilat to ramipril occurs in the kidney (Table 3). Reducing renal function will produce a small reduction in plasma ramipril since it will shift some of the metabolism of ramiprilat from the kidney where 100% is converted to ramipril, to the liver where only about 25% (Fr_L_, Table 3) is converted to ramipril. The second effect of a reduction in a renal function is a proportional reduction in systemic clearance of ramiprilat. The summation of the two effects can be seen in fig. 21 – with 50% and 75% reductions in renal function producing an increase of 1.46 and 2.04 in the peak ramiprilat concentration on the 4^th ^day (approximately steady state). This increase for a 75% reduction is similar to the increase on day 12 observed by Schunkert et. al. [68] in patients with similar reductions in creatinine clearance.

Although it has been known for many years that ACE contains two functionally distinct and catalytically active sites, little attention has been paid to the clinical implications of this. As this PBPK analysis demonstrates, the quantitative clinical time and dosage dependence of the ACE inhibition for a particular ACE inhibitor can be predicted if the in vitro binding kinetics of the two sites for that ACE inhibitor are known. Surprisingly, no kinetic binding measurements at physiological conditions (37°C and 100 mM NaCl) are available for any ACE inhibitor. Probably the single most important implication of this PBPK analysis is to emphasize the importance of the measurement of these in vitro binding constants.

## Conclusion

Because of the high affinity tight binding of the ACE inhibitors and the presence of two (N and C) ACE binding sites with different substrate specificity, the standard ACE assay cannot be directly related to the in vivo inhibition of the renin-angiotensin system. The error in the assay depends critically on a number of factors that are currently not known, such as the inhibitor and substrate binding constants, the catalytic activity of the two ACE sites for the assay substrate and possible interaction between sites. Thus, there are no reliable measurements of the time dependence of the physiological plasma ACE activity for any ACE inhibitor. This PBPK model provides the first quantitative predictions of the pharmacokinetics of the plasma and tissue ACE inhibition of the individual N and C binding sites. Its main limitation is that it is assumed that the N and C site in vitro binding constants determined at 4°C and 300 mM NaCl are valid at physiological conditions.

## Competing interests

The author(s) declare that they have no competing interests.

## Authors' contributions

D. G. L. performed the PBPK model development; the fitting of the PBPK parameters; and the analysis of the results.

R. C. S. provided the experimental data and critically evaluated the manuscript.

## Pre-publication history

The pre-publication history for this paper can be accessed here:



## Supplementary Material

Additional File 1 Section I: Detailed derivation of mathematical description of slow tight binding solutes. Section II: Derivation of relationship between 60 minute ACE assay and true in vivo ACE activity. Section III: Analysis of the sensitivity of the model predictions to parameter variations for IV ramiprilat. Section IV: Analysis of the sensitivity of the model predictions to parameter variations for IV and oral ramipril. Click here for file

## References

[B1] Levitt DG (2002). PKQuest: capillary permeability limitation and plasma protein binding - application to human inulin, dicloxacillin and ceftriaxone pharmacokinetics. BMC Clin Pharmacol.

[B2] Levitt DG (2002). PKQuest: volatile solutes - application to enflurane, nitrous oxide, halothane, methoxyflurane and toluene pharmacokinetics. BMC Anesthesiol.

[B3] Levitt DG (2002). PKQuest: measurement of intestinal absorption and first pass metabolism - application to human ethanol pharmacokinetics. BMC Clin Pharmacol.

[B4] Levitt DG (2002). PKQuest: a general physiologically based pharmacokinetic model. Introduction and application to propranolol. BMC Clin Pharmacol.

[B5] Levitt DG (2003). The use of a physiologically based pharmacokinetic model to evaluate deconvolution measurements of systemic absorption. BMC Clin Pharmacol.

[B6] Levitt DG (2003). The pharmacokinetics of the interstitial space in humans. BMC Clin Pharmacol.

[B7] Levitt DG (2004). Physiologically based pharmacokinetic modeling of arterial - antecubital vein concentration difference. BMC Clin Pharmacol.

[B8] Levitt DG, Schnider TW (2005). Human physiologically based pharmacokinetic model for propofol. BMC Anesthesiol.

[B9] Dzau VJ, Bernstein K, Celermajer D, Cohen J, Dahlof B, Deanfield J, Diez J, Drexler H, Ferrari R, van Gilst W, Hansson L, Hornig B, Husain A, Johnston C, Lazar H, Lonn E, Luscher T, Mancini J, Mimran A, Pepine C, Rabelink T, Remme W, Ruilope L, Ruzicka M, Schunkert H, Swedberg K, Unger T, Vaughan D, Weber M (2001). The relevance of tissue angiotensin-converting enzyme: manifestations in mechanistic and endpoint data. Am J Cardiol.

[B10] Pinto YM, van Veldhuisen DJ, Tjon-Ka-Jie RT, Rooks G, Netzer T, Lie KI (1996). Dose-finding study of imidapril, a novel angiotensin converting enzyme inhibitor, in patients with stable chronic heart failure. Eur J Clin Pharmacol.

[B11] Edling O, Bao G, Feelisch M, Unger T, Gohlke P (1995). Moexipril, a new angiotensin-converting enzyme (ACE) inhibitor: pharmacological characterization and comparison with enalapril. J Pharmacol Exp Ther.

[B12] Chevillard C, Brown NL, Mathieu MN, Laliberte F, Worcel M (1988). Differential effects of oral trandolapril and enalapril on rat tissue angiotensin-converting enzyme. Eur J Pharmacol.

[B13] Roffman DS (2004). High-versus low-dose ACE inhibitor therapy in chronic heart failure. Ann Pharmacother.

[B14] Shapiro R, Riordan JF (1984). Inhibition of angiotensin converting enzyme: dependence on chloride. Biochemistry.

[B15] Bull HG, Thornberry NA, Cordes MH, Patchett AA, Cordes EH (1985). Inhibition of rabbit lung angiotensin-converting enzyme by N alpha-[(S)-1-carboxy-3-phenylpropyl]L-alanyl-L-proline and N alpha-[(S)-1-carboxy-3-phenylpropyl]L-lysyl-L-proline. J Biol Chem.

[B16] Goli UB, Galardy RE (1986). Kinetics of slow, tight-binding inhibitors of angiotensin converting enzyme. Biochemistry.

[B17] Ryan JW, Chung AY, Berryer P, Murray MA, Ryan JP (1986). Slow tight binding inhibitors of angiotensin converting enzyme. Adv Exp Med Biol.

[B18] Bunning P (1987). Kinetic properties of the angiotensin converting enzyme inhibitor ramiprilat. J Cardiovasc Pharmacol.

[B19] Wei L, Clauser E, Alhenc-Gelas F, Corvol P (1992). The two homologous domains of human angiotensin I-converting enzyme interact differently with competitive inhibitors. J Biol Chem.

[B20] Skirgello OE, Binevski PV, Pozdnev VF, Kost OA (2005). Kinetic probes for interdomain cooperation in human somatic angiotensin-converting enzyme. Biochem J.

[B21] Deddish PA, Wang LX, Jackman HL, Michel B, Wang J, Skidgel RA, Erdos EG (1996). Single-domain angiotensin I converting enzyme (kininase II): characterization and properties. J Pharmacol Exp Ther.

[B22] Skoglof A, Gothe PO, Deinum J (1990). Effect of temperature and chloride on steady-state inhibition of angiotensin I-converting enzyme by enalaprilat and ramiprilat. Biochem J.

[B23] Jackson B, Cubela R, Johnston C (1986). Angiotensin converting enzyme (ACE), characterization by 125I-MK351A binding studies of plasma and tissue ACE during variation of salt status in the rat. J Hypertens.

[B24] Georgiadis D, Beau F, Czarny B, Cotton J, Yiotakis A, Dive V (2003). Roles of the two active sites of somatic angiotensin-converting enzyme in the cleavage of angiotensin I and bradykinin: insights from selective inhibitors. Circ Res.

[B25] Bevilacqua M, Vago T, Rogolino A, Conci F, Santoli E, Norbiato G (1996). Affinity of angiotensin I-converting enzyme (ACE) inhibitors for N- and C-binding sites of human ACE is different in heart, lung, arteries, and veins. J Cardiovasc Pharmacol.

[B26] Fabris B, Chen BZ, Pupic V, Perich R, Johnston CI (1990). Inhibition of angiotensin-converting enzyme (ACE) in plasma and tissue. J Cardiovasc Pharmacol.

[B27] Johnston CI, Fabris B, Yamada H, Mendelsohn FA, Cubela R, Sivell D, Jackson B (1989). Comparative studies of tissue inhibition by angiotensin converting enzyme inhibitors. J Hypertens Suppl.

[B28] Beldent V, Michaud A, Wei L, Chauvet MT, Corvol P (1993). Proteolytic release of human angiotensin-converting enzyme. Localization of the cleavage site. J Biol Chem.

[B29] Rigat B, Hubert C, Alhenc-Gelas F, Cambien F, Corvol P, Soubrier F (1990). An insertion/deletion polymorphism in the angiotensin I-converting enzyme gene accounting for half the variance of serum enzyme levels. J Clin Invest.

[B30] King JN, Maurer M, Toutain PL (2003). Pharmacokinetic/pharmacodynamic modelling of the disposition and effect of benazepril and benazeprilat in cats. J Vet Pharmacol Ther.

[B31] Toutain PL, Lefebvre HP, King JN (2000). Benazeprilat disposition and effect in dogs revisited with a pharmacokinetic/pharmacodynamic modeling approach. J Pharmacol Exp Ther.

[B32] Toutain PL, Lefebvre HP (2004). Pharmacokinetics and pharmacokinetic/pharmacodynamic relationships for angiotensin-converting enzyme inhibitors. J Vet Pharmacol Ther.

[B33] Francis RJ, Brown AN, Kler L, Fasanella d'Amore T, Nussberger J, Waeber B, Brunner HR (1987). Pharmacokinetics of the converting enzyme inhibitor cilazapril in normal volunteers and the relationship to enzyme inhibition: development of a mathematical model. J Cardiovasc Pharmacol.

[B34] Lees KR, Kelman AW, Reid JL, Whiting B (1989). Pharmacokinetics of an ACE inhibitor, S-9780, in man: evidence of tissue binding. J Pharmacokinet Biopharm.

[B35] Brockmeier D (1995). Tight binding of ramiprilat to ACE: consequences for pharmacokinetic and pharmacodynamic measurements. Int J Clin Pharmacol Ther.

[B36] Brockmeier D (1998). Tight binding influencing the future of pharmacokinetics. Methods Find Exp Clin Pharmacol.

[B37] Levitt DG PKQuest: One stop pharmacokinetic program.. http://www.pkquest.com.

[B38] van Griensven JM, Schoemaker RC, Cohen AF, Luus HG, Seibert-Grafe M, Rothig HJ (1995). Pharmacokinetics, pharmacodynamics and bioavailability of the ACE inhibitor ramipril. Eur J Clin Pharmacol.

[B39] Thuillez C, Richer C, Giudicelli JF (1987). Pharmacokinetics, converting enzyme inhibition and peripheral arterial hemodynamics of ramipril in healthy volunteers. Am J Cardiol.

[B40] Zeitz CJ, Campbell DJ, Horowitz JD (2003). Myocardial uptake and biochemical and hemodynamic effects of ACE inhibitors in humans. Hypertension.

[B41] Eckert HG, Badian MJ, Gantz D, Kellner HM, Volz M (1984). Pharmacokinetics and biotransformation of 2-[N-[(S)-1-ethoxycarbonyl-3-phenylpropyl]-L-alanyl]-(1S,3S, 5S)-2-azabicyclo [3.3.0]octane-3-carboxylic acid (Hoe 498) in rat, dog and man. Arzneimittelforschung.

[B42] Ranadive SA, Chen AX, Serajuddin AT (1992). Relative lipophilicities and structural-pharmacological considerations of various angiotensin-converting enzyme (ACE) inhibitors. Pharm Res.

[B43] Abu-Zahra TN, Wolkoff AW, Kim RB, Pang KS (2000). Uptake of enalapril and expression of organic anion transporting polypeptide 1 in zonal, isolated rat hepatocytes. Drug Metab Dispos.

[B44] Liu L, Pang KS (2005). The roles of transporters and enzymes in hepatic drug processing. Drug Metab Dispos.

[B45] de Lannoy IA, Barker F, Pang KS (1993). Formed and preformed metabolite excretion clearances in liver, a metabolite formation organ: studies on enalapril and enalaprilat in the single-pass and recirculating perfused rat liver. J Pharmacokinet Biopharm.

[B46] de Lannoy IA, Hirayama H, Pang KS (1990). A physiological model for renal drug metabolism: enalapril esterolysis to enalaprilat in the isolated perfused rat kidney. J Pharmacokinet Biopharm.

[B47] de Lannoy IA, Pang KS (1993). Combined recirculation of the rat liver and kidney: studies with enalapril and enalaprilat. J Pharmacokinet Biopharm.

[B48] Pang KS, Cherry WF, Ulm EH (1985). Disposition of enalapril in the perfused rat intestine-liver preparation: absorption, metabolism and first-pass effect. J Pharmacol Exp Ther.

[B49] Sirianni GL, Pang KS (1998). Intracellular and not intraluminal esterolysis of enalapril in kidney. Studies with the single pass perfused nonfiltering rat kidney. Drug Metab Dispos.

[B50] Friedman DI, Amidon GL (1989). Passive and carrier-mediated intestinal absorption components of two angiotensin converting enzyme (ACE) inhibitor prodrugs in rats: enalapril and fosinopril. Pharm Res.

[B51] Morrison RA, Chong S, Marino AM, Wasserman MA, Timmins P, Moore VA, Irwin WJ (1996). Suitability of enalapril as a probe of the dipeptide transporter system: in vitro and in vivo studies. Pharm Res.

[B52] Alhenc-Gelas F, Weare JA, Johnson RLJ, Erdos EG (1983). Measurement of human converting enzyme level by direct radioimmunoassay. J Lab Clin Med.

[B53] Hattori MA, Del Ben GL, Carmona AK, Casarini DE (2000). Angiotensin I-converting enzyme isoforms (high and low molecular weight) in urine of premature and full-term infants. Hypertension.

[B54] Cushman DW, Cheung HS (1971). Concentrations of angiotensin-converting enzyme in tissues of the rat. Biochim Biophys Acta.

[B55] Hwang DR, Eckelman WC, Mathias CJ, Petrillo EWJ, Lloyd J, Welch MJ (1991). Positron-labeled angiotensin-converting enzyme (ACE) inhibitor: fluorine-18-fluorocaptopril. Probing the ACE activity in vivo by positron emission tomography. J Nucl Med.

[B56] Morin JP, Moulin B, Borghi H, Fillastre JP (1989). High affinity binding sites for Perindopril a new inhibitor of angiotensin-I-converting enzyme (ACE) in the rabbit kidney: possible evidence for localization of ACE in endothelial structures and in glomerular mesangium. Int J Tissue React.

[B57] Reneland R, Haenni A, Andersson PE, Andren B, Lithell H (1999). Skeletal muscle angiotensin-converting enzyme and its relationship to blood pressure in primary hypertension and healthy elderly men. Blood Press.

[B58] Reneland R, Lithell H (1994). Angiotensin-converting enzyme in human skeletal muscle. A simple in vitro assay of activity in needle biopsy specimens. Scand J Clin Lab Invest.

[B59] Sakaguchi K, Jackson B, Chai SY, Mendelsohn FA, Johnson CI (1988). Effects of perindopril on tissue angiotensin-converting enzyme activity demonstrated by quantitative in vitro autoradiography. J Cardiovasc Pharmacol.

[B60] Sun Y, Diaz-Arias AA, Weber KT (1994). Angiotensin-converting enzyme, bradykinin, and angiotensin II receptor binding in rat skin, tendon, and heart valves: an in vitro, quantitative autoradiographic study. J Lab Clin Med.

[B61] Jonsson JR, Game PA, Head RJ, Frewin DB (1994). The expression and localisation of the angiotensin-converting enzyme mRNA in human adipose tissue. Blood Press.

[B62] Nussberger J, Juillerat L, Perret F, Waeber B, Bellet M, Brunner J, Menard J (1989). Need for plasma angiotensin measurements to investigate converting-enzyme inhibition in humans. Am Heart J.

[B63] Weisser K, Schloos J (1993). Measurements of serum ACE activity in vitro after administration of enalapril in man cannot reflect inhibition of the enzyme in vivo. Methods Find Exp Clin Pharmacol.

[B64] van Griensven JM, Seibert-Grafe M, Schoemaker HC, Frolich M, Cohen AF (1993). The pharmacokinetic and pharmacodynamic interactions of ramipril with propranolol. Eur J Clin Pharmacol.

[B65] Verho M, Luck C, Stelter WJ, Rangoonwala B, Bender N (1995). Pharmacokinetics, metabolism and biliary and urinary excretion of oral ramipril in man. Curr Med Res Opin.

[B66] Wei L, Alhenc-Gelas F, Corvol P, Clauser E (1991). The two homologous domains of human angiotensin I-converting enzyme are both catalytically active. J Biol Chem.

[B67] Nordstrom M, Abrahamsson T, Ervik M, Forshult E, Regardh CG (1993). Central nervous and systemic kinetics of ramipril and ramiprilat in the conscious dog. J Pharmacol Exp Ther.

[B68] Schunkert H, Kindler J, Gassmann M, Lahn W, Irmisch R, Ritz E, Debusmann ER, Pujadas JO, Koch KM, Sieberth HG (1989). Pharmacokinetics of ramipril in hypertensive patients with renal insufficiency. Eur J Clin Pharmacol.

[B69] Shionoiri H, Miyakawa T, Yasuda G, Ishikawa Y, Umemura S, Miyajima E, Kaneko Y (1987). Pharmacokinetics of a single dose of ramipril in patients with renal dysfunction: comparison with essential hypertension. J Cardiovasc Pharmacol.

[B70] Aurell M, Delin K, Herlitz H, Ljungman S, Witte PU, Irmisch R (1987). Pharmacokinetics and pharmacodynamics of ramipril in renal failure. Am J Cardiol.

[B71] Debusmann ER, Pujadas JO, Lahn W, Irmisch R, Jane F, Kuan TS, Mora J, Walter U, Eckert HG, Hajdu P, Metzger H (1987). Influence of renal function on the pharmacokinetics of ramipril (HOE 498). Am J Cardiol.

[B72] Jaspard E, Wei L, Alhenc-Gelas F (1993). Differences in the properties and enzymatic specificities of the two active sites of angiotensin I-converting enzyme (kininase II). Studies with bradykinin and other natural peptides. J Biol Chem.

[B73] Heintz B, Verho M, Brockmeier D, Luckel G, Maigatter S, Sieberth HG, Rangoonwala B, Bender N (1993). Multiple-dose pharmacokinetics of ramipril in patients with chronic congestive heart failure. J Cardiovasc Pharmacol.

