# Supplementary material for: Human physiologically based pharmacokinetic model for ACE inhibitors: ramipril and ramiprilat
Source: BMC Clin Pharmacol. 2006 Jan 6;6:1. doi: 10.1186/1472-6904-6-1 (PMC1373666; doi:10.1186/1472-6904-6-1)
Supplement: Additional File 1 — Section I: Detailed derivation of mathematical description of slow tight binding solutes. Section II: Derivation of relationship between 60 minute ACE assay and true in vivo ACE activity. Section III: Analysis of the sensitivity of the model predictions to parameter variations for IV ramiprilat. Section IV: Analysis of the sensitivity of the model predictions to parameter variations for IV and oral ramipril. [file 1472-6904-6-1-S1.doc]

***ACE_supplemental: Additional material for manuscript:***

**Human physiologically based pharmacokinetic model for ACE inhibitors: ramipril and ramiprilat David G. Levitt and Rik C. Schoemaker**

**I. Derivation of mathematical description for slow, tight binding solutes.**

Notation:

The notation used here is slightly different from that in the body of paper. Lower case letters refer to the free concentration, and upper case to the total concentration.

C total ramiprilat concentration.

c free ramiprilat concentration.

cL, cT, cp loosely ACE bound, tightly ACE bound and albumin bound concentration.

E, e total and unbound ACE concentration.

k1, k2 rate constants for formation of the tight ACE complex.

KL, Kt equilibrium dissociation constant for loose and total ACE binding.

B, Pi Albumin binding constant and albumin tissue concentration.

In the original PKQuest derivation it was assumed that, for each solute s and tissue i, the relationship between the free, unbound tissue concentration (ci) and the bound concentration (cb) was described by an equilibrium tissue binding constant (Kt):

where e is the concentration of free binding sites. For slow, tight binding solutes the binding is not at equilibrium and is described, in general, by a two step process in which there is rapid equilibration to form a “loosely” bound form (cL), followed by slow conformational change to a “tightly” bound form (cT):

In general, the binding is characterized by 3 constants: the equilibrium “loose” binding dissociation constant KL and the rate constants k1 and k2 characterizing the time dependent slow conversion between a loose and “tight” binding form. At equilibrium, the relation between the total bound (= cL+cT) and free concentration is described by the dissociation constant Kt:

$$c_{b}= c_{L}+c_{T}=ce/K_{t} K_{t}=K_{L}/(1+k_{1}/k_{2})$$

For the ACE inhibitors, it has been shown that the tight binding can be described as a single step process and the loose binding concentration (cl) is negligible relative to ct [1, 2]. This limit is implemented in PKQuest by setting KL to an arbitrary, very large value (so that cL  0) and k1 then becomes a function of KL (which is known) and the two parameters Kt and k2 (eq. ):

$$k_{1}=k_{2}(K_{L}/K_{t}-1)$$

In this limit, which is used for the modeling of ACE binding, the tight binding is characterized by two constants: the unbinding rate constant k2 and the tight binding dissociation constant Kt. In the following derivation, the general two step tight binding process will be described.

The ACE inhibitors are also bound non-specifically to plasma and tissue proteins, primarily albumin. The albumin bound component (cb) is proportional to the extracellular albumin concentration in tissue i (Pi):

$$c_{p}=B P_{i} c$$

For example, for ramiprilat, 56% is bound in human plasma [3]:

$$c_{p}/(c_{p}+c)=0.56 \Rightarrow B P_{plasma}=c_{p}/c=1.28$$

For tissue i:

$$BP_{i}=(P_{i}/P_{plasma}) BP_{plasma}$$

Using the previously determined PKQuest “standard human” extracellular albumin concentrations in the different tissues (Pi) [4], the value of albumin binding in the tissues can be determined simply by inputting the experimental value for the fraction bound in plasma.

Using the notation from the original PKQuest paper [5], the mass balance for tissue i is described by:

$$v_{i}\frac{dC_{i}}{dt}=\rho_{B}F_{i}(C_{A}-C_{V})+cinput_{i}-Q_{i}$$

Where Ci is the total concentration, vi is the volume, F is the blood flow, CA and CV are the blood concentrations entering and leaving the tissue, cinput is the local input to the tissue, and Q is the rate of metabolism (see [5] for a detailed definition of the notation). The total concentration Ci is the sum of 4 terms: the free concentration c, the albumin bound concentration cp, the loosely ACE bound cL and the tightly ACE bound concentration cT. The left hand side of eq. can be written in terms of these 4 concentrations:

$$v\frac{dC}{dt}=v wfract \frac{d}{dt}(c+c_{p}+c_{L}+c_{T})=v wfract\frac{d}{dt}[c(1+BP+e/K_{L})+c_{T}]$$

The total tight binding enzyme concentration E is defined by:

$$E=e+c_{l}+c_{t}$$

The derivatives of ct and e are described by:

$$\begin{matrix} \frac{d c_{t}}{dt}=k_{1}c_{l}-k_{2}c_{t}=(k_{1}/K_{L})c e-k_{2}c_{t} \\ \frac{de}{dt}= -\frac{d c_{t}}{dt}/(1+c/K_{L}) - (E-c_{t})(\frac{1}{K_{L}}\frac{dc}{dt})/(1+c/K_{L})^{2} \end{matrix}$$

Substituting eq. in eq. , the left hand side of eq. can be written in the form:

$$v\frac{dC}{dt}=v wfract\frac{dc}{dt}[(1+BP+e/K_{L})-(E-c_{t})\frac{c}{K_{L}^{2}(1+c/K_{L})^{2}}] +\frac{d c_{t}}{dt}[\frac{K_{L}}{K_{L}+c}]$$

The (CA-CV) term on the right hand side of eq. can be expanded in the form:

Note that it is assumed that the tight concentration in the blood leaving the tissue (ctV) equals the tight concentration entering (ctA), based on the assumption that the exchange is so slow that there is no significant equilibration during one capillary transit time. For all tissues except the lung, the entering concentration equals the central arterial concentration. For the lung, the entering concentration equals the central venous concentration. The venous (cV) concentration depends on the arterial concentration (cA), and the rate of exchange between the free tissue concentration (c) and the capillary, which is determine by permeability parameter fclear (see [5] for details:

$$fclear=(c_{A}-c_{V})/(c_{A}-c)$$

The PKQuest parameter fclear can be related to the capillary permeability – surface area product ([6].

Substituting and into yields the final tight binding differential equation for the concentration in tissue i. The independent variables are the free tissue concentration c and the tightly bound concentration cT. A similar set of equations is used for the central venous and arterial compartments. There is an equation of this form for each tissue, with each tissue i having a different free and bound concentration and different values for the total tight binding ACE concentration (Ei) and the albumin concentration (Pi). The ACE rate constants KL, k1 and k2 are the same for all tissues. The implementation in PKQuest allows for an arbitrary number (Nt) of independent tight binding proteins, with each protein j (j=1..Nt) and tissue i characterized by Eij, KLj, k1j and k2j. For the ACE inhibitors discussed in the text, it is assumed that there are two, non-interacting, tight binding protein sites (representing C terminal and N terminal ACE binding sites) with equal concentrations (Ei1 = Ei2).

For each tissue i and solute j there is a differential equation for the each free concentration (dcij/dt, eqs. and ) and for each tightly bound concentration (dctij/dt, j = 1..Nt) ( eq. ). In addition, another differential equation (see eq. 5, main text) must be added for each tissue that has a finite cell membrane permeability (e.g. ramipril). The system of differential equations is solved using the Maple numerical solver ([www.maplesoft.com](http://www.maplesoft.com/)). These modifications have been added to the PKQuest software routine. The complete software code is freely available at [www.PKQuest.com](http://www.PKQuest.com/).

**II. Derivation of relationship between 60 minute ACE assay and true in vivo ACE activity.**

In the following analysis it is assumed that the two sites are independent. For one site (either C or N):

Where I , S and E are the free inhibitor, substrate and ACE enzyme concentrations, IE and SE are the concentrations of the enzyme inhibitor and enzyme substrate complexes, and Et and It are the total ACE and inhibitor concentration. It is assumed that the substrate concentration is much greater than Et or It and, therefore, S is constant, equal to the fixed assay concentration. Solving eq. for E:

$$E = (Et-I_{E})/(1+S/K_{S})$$

The experimental enzyme activity in the presence of inhibitor is described by:

$$Activity=k_{cat}S_{E} = k_{cat}S E/K_{S} = k_{cat}S(Et-I_{E})/(K_{S}+S)$$

The activity in the absence of inhibitor is:

$$Activity_{0}= k_{cat}S Et/(K_{S}+S)$$

The assay involves diluting the plasma in the assay solution and measuring the total activity for 60 minutes. During this 60 minute, the inhibitor dissociates from the enzyme because of dilution and the concentration of the enzyme inhibitor complex changes with time (IE(t)). The experimental enzyme activity (Assay60) is defined as the total activity during a 60 minute incubation relative to the activity for the same dilution conditions in the absence of inhibitor:

$$Assay_{60}=\int_{0}^{60} Activity(t)dt /(60 Activity_{0})=(60Et)^{-1}\int_{0}^{60} [Et-I_{E}(t)]dt$$

The value of IE(t) is determined from a solution of the differential equation for enzyme inhibitor binding:

$$d I_{E}(t)/dt = k_{1}I E - k_{-1}I_{E}(t)$$

As an initial condition (IE(t=0)) for this differential equation it is assumed that the enzyme and inhibitor are at equilibrium. This initial condition also corresponds to the true “in vivo” enzyme activity, without any dilution artifacts. Figure 15 of the main text describes the fractional change in IE(t) during the 60 minute Vertex assay. Figure 16 shows the relationship between the “in vivo” and the 60 minute assay for varying concentrations of the plasma ramiprilat.

**III. Sensitivity of the model predictions to parameter variations for IV ramiprilat.**

This section describes a detailed analysis of the dependence of the model predictions on variations in the model parameters. The focus of the analysis is a series of figures describing the model predictions for a range of parameter values, compared with the experimental data for IV ramiprilat for subject 4. In each figure the black line corresponds to the model result using the “standard” parameters described in body of the paper and the colored lines are for variations of the parameters. The left panel show the results at short times (0 to 440 min) and the right panel is for the entire length of the experimental measurements (0 to 4410 min).

Figure S1 shows the influence of variations in Clu, the intrinsic (“unbound”) renal clearance. The black line is for Clu = 0.4 liters/min, the value that gave the optimal fit to the experimental data for subject 4 (black squares). The red and green lines are the model concentrations for a 33% increase or decrease in renal clearance. Figures S2 and S3 describe the effect of varying the dissociation constants (KN, KC) or the unbinding rate constants (k-N, k-C), for the two ACE binding sites. Figures S4 and S5 show the effect of variations in either the plasma or tissue ACE concentration, respectively. When the tissue ACE was varied, the concentration was increased by the same factor in all the tissues.

At early times (< 300 minutes, left panels, figs. S1 – S5) the plasma ramiprilat is much greater than the plasma ACE (= 1.65 nM ), the fraction of ramiprilat that is bound to ACE is negligible, and the only parameter that influences the plasma ramiprilat level is the renal clearance (fig. S1). The parameters that are related to ACE binding (plasma and tissue ACE and the binding constants, figs S2 – S5) only become important at long times when the plasma ramiprilat approaches the plasma ACE concentration. As shown in fig. S3, there is no significant change in the plasma ramiprilat if the unbinding rate constant (k2) is increased to infinity, which is equivalent to assuming a rapid equilibrium binding. It is surprising that the C site unbinding rate constant of 0.00168/min (time constant = 9.92 hours) does not influence the kinetics. The reason is that for the IV ramiprilat input the ACE binding does not become important until after about 10 hours, a time comparable to the rate constant. As will be shown below (fig. S13), for oral ramipril input the plasma ramiprilat concentration is much lower at early times and the value of k2 does have a small but significant influence on the kinetics.

As seen in figs S2, S4 and S5, variations in Kt, plasma ACE and tissue ACE have similar effects on the shape of the plasma ramiprilat curve and there are many different combinations of these parameters that provide equally good fits to the experimental data. The experimental renal ramiprilat excretion can be used to distinguish between these different combinations. Since the renal clearance acts only on the unbound ramiprilat, it provides a direct measure of the fraction of ramiprilat that is unbound. Figure S6 provides two examples of the use of the urinary renal excretion to distinguish between different combinations of the parameters. The top row shows the plasma ramiprilat and urinary excretion for the “standard” parameters. It can be seen that there is good agreement between the experimental data (red squares) and model predictions (black squares). The middle row shows the results for the case where plasma ACE and tissue ACE have been multiplied by a factor of 1.25 and 0.4, respectively. It can be seen that although this change provides as good a fit to the plasma ramiprilat as the standard set, the resulting model urine ramiprilat is significantly less than the experimental value. Similarly (bottom panel), although scaling plasma ACE by 1.25 and KN and KC by 3.0 fits the plasma ramiprilat, the predicted excretion is now greater than the experimental.

As this analysis for subject 4 indicates, the combination of ramiprilat plasma concentration and urinary excretion data provides a unique determination of these PBPK ramiprilat parameters. It has been assumed in this analysis that renal clearance is the only pathway for ramiprilat removal. Subject 4 was chosen because the total urinary recovery of ramiprilat (2.3 mg) was approximately equal to the total amount infused (2.68 mg) and the collection was extended out to 72 hours, when only about 3% should remain in the body. For all subjects (extrapolating to 72 hours) the ramiprilat recovery varied from 57 to 137% with a mean of 82%.

The ramiprilat binding constants cannot be uniquely determined by this data and only qualitative constraints can be placed on them. As illustrated in fig. S3, any value of k-N and k-Cranging from 10% of the standard value, up to infinity provide similar fits. In addition, one can modify the relative values of KN and KC and still fit all the data. For example, using an identical K of 0.140 nM for both sites provides an equally good fit to the data (not shown). This value of K is identical to that reported by Brockmeier using a single binding site assumption [7, 8].

**IV. Sensitivity of the model predictions to parameter variations for IV and oral ramipril.**

This section describes an analysis of the parameter dependence of the model predictions of the plasma ramipril and ramiprilat following IV and oral ramipril. The ramiprilat kinetics are described by the 5 parameters discussed in section II. Seven parameters are required to model the IV ramipril experiments (see Methods): Clint_L, Clint_K and FrL describe the liver and kidney metabolism of ramipril and the conversion to ramiprilat; PsL and PsK describe the rate that this ramiprilat leaves the liver and kidney and enters the systemic circulation; PsT describes the rate that ramipril enters the intracellular water of the peripheral tissues and fu_cell describes the intracellular fraction unbound which determines the ramipril equilibrium volume of distribution. Five additional parameters (AD, a, T, AR, Arec), are used to model the intestinal ramipril absorption that is necessary to describe the oral ramipril experiments.

In total, 12 new parameters are required to describe the oral and IV ramipril data. Given this large number, it is clearly not possible to specify a unique, quantitative data set. However, the following investigation of the sensitivity of the model fits to the experimental data indicates that all of these parameters contribute significantly to model predictions.

**Adjustable liver and kidney ramipril metabolism (Clint_L, Clint_K, and FrL).**  The requirement for these two independent metabolic components arises from the different way the liver and kidney act on an IV versus an oral input. The PBPK model assumes that a fraction of the total liver ramipril metabolism is converted to ramiprilat (which enters the systemic circulation) while all the renal metabolism of ramipril is converted to systemic ramiprilat. This effect combined with the first pass metabolism for an oral input implies that the fraction of an oral input that is converted to systemic ramiprilat will be less than that of an IV input. Thus, these two independent metabolic components provide an extra degree of freedom in the PBPK model that improves the fit to the IV and oral ramipril input. For example, for subject 4, the metabolic parameters are: 1) Clint_L = 3.6 l/min (the intrinsic liver clearance); 2) Clint_K = 0.0 l/min (the intrinsic kidney clearance); and 3) FrL = 0.35 (the fraction of the liver metabolism that is converted to systemic ramiprilat). Since these parameters are “intrinsic clearances” and are a function of the intracellular concentrations, they are only indirectly related to the actual liver and kidney clearance. By directly integrating the model organ metabolism, the actual total clearance can be determined. For subject 4, the fraction of total ramiprilat metabolized by the kidney is 27% for an IV input and 14% for an oral input, and, by assumption, all of this is converted to ramiprilat. The fraction of total ramiprilat metabolized by the liver is 63% for an IV input and 86% for an oral input and 35% (=FrL) of this is converted to ramiprilat. Thus, 52% of the IV dose versus 44% of an oral dose of ramipril is converted to ramiprilat. This difference increases as FrR decreases and frkid increases. Figure S7 shows the optimal fit that can be obtained for subject 4 data if there were no kidney metabolism. The model fit is clearly better than when both metabolic components are present (compare figs. 6 (main text) and S7).

**Liver and kidney ramiprilat cell membrane permeability (Psliver and Pskidney).** The rate the ramiprilat formed from ramipril leaves the liver cell and enters the systemic circulation depends on the product fu­_cell x Ps (see eq. 5, main text) where fu_cell is the intracellular unbound fraction and is a measure of intracellular binding and Ps is the cell membrane permeability. In the model, fu_cell was set to an arbitrary fixed value, and Ps is the primary parameter determining the rate of appearance of ramiprilat in the systemic circulation. Figure S8 demonstrates the influence of the value of the liver ramiprilat Ps on the plasma ramiprilat concentration following oral ramipril. The value of Ps for the kidney has a similar effect.

**Finite ramipril cell membrane permeability (PsT).** A cell membrane permeability (Ps) of zero means that the ramipril is restricted to the extracellular space, while a finite cell membrane permeability allows the ramipril to distribute in all the body water, with a larger volume of distribution. Figure S9 shows the influence of the value of tissue Ps on the plasma ramipril following an IV ramipril input. (All the non liver and kidney tissues are assumed to have the same Ps value). It can be seen that a finite cell permeability is necessary, and any value greater than 0.05/min provides an equally good fit.

**Slow intestinal absorption component (Arec).** Five parameters are required to describe the intestinal ramipril absorption. The major component is the 3 parameter (A, a and T) ramipril gamma distribution function where A is the total amount absorbed. This component peaks at about 30 minutes and drops to 0 by about 200 minutes (see fig. 14). In 6 of the 11 subjects (Table 4) a second, much slower absorption component that extends out to 3000 minutes (about 2 days) is required. The total amount absorbed in this second component (=Arec) varies from 0 to 30% of that in the primary absorption (Table 4). Figure S10 shows the influence of this second component on the plasma ramipril and ramiprilat for subject 4. The absorption rate of the second component is only about 1% of the rate of the major component and the total amount absorbed by the second component is 23% of that of the major component. This component has no detectable affect on the plasma ramipril (fig. S10) because the change produced is far below the analytical detection limits.

**Direct intestinal ramiprilat absorption (AR).** There is a third model component of oral ramipril absorption in which the ramipril is converted to ramiprilat either in the intestinal lumen or epithelial cell and directly absorbed. Since this component directly adds to the systemic ramiprilat, it produces a much faster rise in plasma ramiprilat than the other two ramipril absorption components. For simplicity, it is assumed that this component has the same time course as the major ramipril component (i.e. same a and T) with a molar amount equal to AR. This component varies from 0 to 13% of the major component (ave. = 6%) (Table 4). For subject 4 this component is less than 2% of the major component and is not significant. However, in other subjects, it has a more important effect. Figure S11 shows the experimental data for subject 12. After a bolus IV ramipril infusion, the plasma ramiprilat peaks at about 300 minutes, 295 minutes after the plasma ramipril peak. This delay results from the liver and kidney ramipril metabolism and transport of ramiprilat from the cell to the systemic circulation. One would expect that there should be similar delays after oral ramipril and the plasma ramiprilat should peak at about 360 minutes (300 minutes after the ramipril peak at 60 minutes for oral input). However, the ramiprilat after oral ramipril actually peaks at 118 minutes, much earlier than the ramiprilat peak for the bolus IV ramipril input. The only possible explanation for this early peak is that some of the intestinally absorbed ramipril is directly converted to ramiprilat, bypassing the necessity or liver or renal metabolism. Figure S12 shows the model predictions for subject 12, with and without the addition of this third, direct, ramiprilat absorption component (equal to 14.8% of the major component, Table 4).

**Dependence on unbinding rate constant.**  As described above (see fig.S3), for the IV ramiprilat input the kinetics are relatively independent of the unbinding rate constants (k-C and k-N) because of the long time before the concentration reaches low enough levels that binding becomes important. In contrast, for the oral ramipril input the peak ramiprilat concentration is much lower and the binding kinetics have a more important influence. Figure S13 shows the model results for the oral ramipril input for subject 4 with 3 different values of kC and kN (the standard values, and a 10 fold increase or decrease). Although there are significant differences, they are relatively small compared to the other uncertainties in the model.

**Figure Legends:**

Figure S1. Effect of variations in the model intrinsic (unbound) renal clearance (Clu) on the PBPK model plasma ramiprilat following IV ramiprilat for subject 4 at early times (left) and for the entire time course (right). The open squares are the experimental plasma ramiprilat values.

Figure S2. Effect of variations in the equilibrium dissociation constants (KN and KC) on the PBPK model plasma ramiprilat following IV ramiprilat for subject 4 at early times (left) and for the entire time course (right). The open squares are the experimental plasma ramiprilat values.

Figure S3. Effect of variations in the unbinding rate constants (k-N and k-C) on the PBPK model plasma ramiprilat following IV ramiprilat for subject 4 at early times (left) and for the entire time course (right). The open squares are the experimental plasma ramiprilat values.

Figure S4. Effect of variations in the plasma ACE concentration on the PBPK model plasma ramiprilat following IV ramiprilat for subject 4 at early times (left) and for the entire time course (right). Since the tissue/plasma ACE ratio is maintained constant, increases in plasma ACE increases tissue ACE by the same fraction. The open squares are the experimental plasma ramiprilat values.

Figure S5. Effect of variations in the tissue/plasma ACE concentration ratio on the PBPK model plasma ramiprilat following IV ramiprilat for subject 4 at early times (left) and for the entire time course (right). The plasma ACE is unchanged and the tissue ACE changes by the same fraction in all tissues. The open squares are the experimental plasma ramiprilat values.

Figure S6. Use of the experimental renal ramiprilat clearance data to distinguish between different combinations of PBPK parameters. Left column plots the plasma ramiprilat (nanomoles/liter) and right column plots the amount of urine ramiprilat (nanomoles) collected in each experimental time period. Top panels: standard, optimal, parameter set. Middle panels: Increasing plasma ACE by 1.25 and decreasing tissue ace by 0.4 results in good fit to plasma ramiprilat (left), but high urine excretion (right). Bottom panels: Increasing plasma ACE by 1.25 and increasing dissociation constant (KN and KC) by 3 results in a good fit to plasma ramiprilat (left), but low urine excretion.

Figure S7. Same plot as fig. 6 (main text) except no renal metabolism. The other parameters have been adjusted to optimize the fit to all the data.

Figure S8. Effect of variations in the liver cell membrane ramiprilat permeability on the PBPK model plasma ramiprilat following oral ramipril for subject 4. The open squares are the experimental plasma ramiprilat values.

Figure S9. Effect of variations in the tissue (not liver or kidney) cell membrane ramipril permeability on the PBPK model plasma ramipril following oral ramipril for subject 4. The open squares are the experimental plasma ramipril values.

Figure S10. Plasma ramipril (top) and ramiprilat (bottom) following oral ramipril for subject 4 with (black) and without (red) the slow intestinal ramipril absorption component.

Figure S11. Experimental data for subject 12. Top panels: plasma ramipril (left) and ramiprilat (right) following IV ramipril. Bottom panels: plasma ramipril (left) and ramiprilat (right) following oral ramipril. The peak in the plasma ramiprilat occurs earlier for the oral ramipril than for the bolus IV ramipril.

Figure S12. PBPK model fit to the experimental data for oral ramipril for subject 12 with (black) and without (red) the direct ramiprilat absorption component.

Figure S13. Effect of variations in the ramiprilat dissociation rate constant for the N and C site on the plasma ramiprilat following oral ramipril in subject 4.

**References:**

1. Deddish PA, Wang LX, Jackman HL, Michel B, Wang J, Skidgel RA, Erdos EG: **Single-domain angiotensin I converting enzyme (kininase II): characterization and properties**. *J Pharmacol Exp Ther* 1996, **279**(3):1582-1589.

2. Wei L, Clauser E, Alhenc-Gelas F, Corvol P: **The two homologous domains of human angiotensin I-converting enzyme interact differently with competitive inhibitors**. *J Biol Chem* 1992, **267**(19):13398-13405.

3. Eckert HG, Badian MJ, Gantz D, Kellner HM, Volz M: **Pharmacokinetics and biotransformation of 2-[N-[(S)-1-ethoxycarbonyl-3-phenylpropyl]-L-alanyl]-(1S,3S, 5S)-2-azabicyclo [3.3.0]octane-3-carboxylic acid (Hoe 498) in rat, dog and man**. *Arzneimittelforschung* 1984, **34**(10B):1435-1447.

4. Levitt DG: **The pharmacokinetics of the interstitial space in humans**. *BMC Clin Pharmacol* 2003, **3**(1):3.

5. Levitt DG: **PKQuest: a general physiologically based pharmacokinetic model. Introduction and application to propranolol**. *BMC Clin Pharmacol* 2002, **2**(1):5.

6. Levitt DG: **PKQuest: capillary permeability limitation and plasma protein binding - application to human inulin, dicloxacillin and ceftriaxone pharmacokinetics**. *BMC Clin Pharmacol* 2002, **2**(1):7.

7. Brockmeier D: **Tight binding of ramiprilat to ACE: consequences for pharmacokinetic and pharmacodynamic measurements**. *Int J Clin Pharmacol Ther* 1995, **33**(12):631-638.

8. Brockmeier D: **Tight binding influencing the future of pharmacokinetics**. *Methods Find Exp Clin Pharmacol* 1998, **20**(6):505-516.

Figure S1. Effect of variations in the model intrinsic (unbound) renal clearance (Clu) on the PBPK model plasma ramiprilat following IV ramiprilat for subject 4 at early times (left) and for the entire time course (right). The open squares are the experimental plasma ramiprilat values.


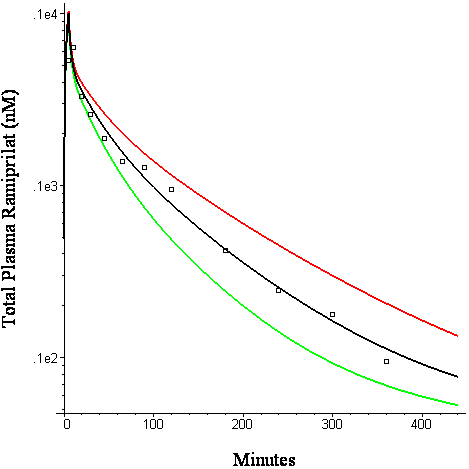

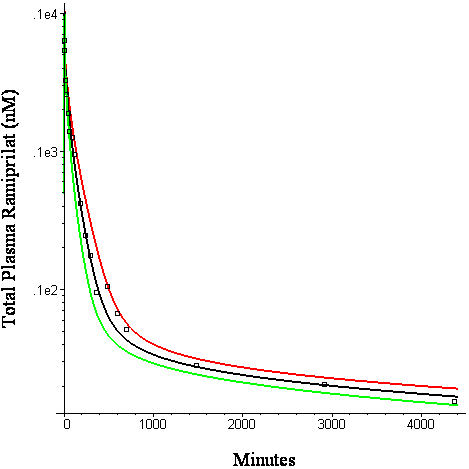


**Clu = 0.3 l/min**

**Clu = 0.4 l/min**

**Clu = 0.533 l/min**

**Subject 4 IV ramiprilat**

Figure S2. Effect of variations in the equilibrium dissociation constants (KN and KC) on the PBPK model plasma ramiprilat following IV ramiprilat for subject 4 at early times (left) and for the entire time course (right). The open squares are the experimental plasma ramiprilat values.


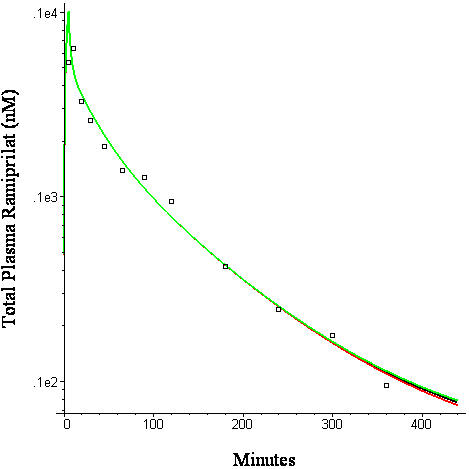

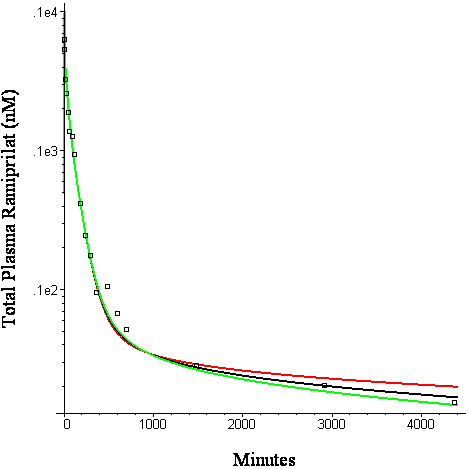


**KN = 0.138 nM KC = 0.019 nM**

**KN = 0.276 nM KC = 0.039 nM**

**KN = 0.414 nM KC = 0.058 nM**

**Subject 4 IV ramiprilat**

Figure S3. Effect of variations in the unbinding rate constants (k-N and k-C) on the PBPK model plasma ramiprilat following IV ramiprilat for subject 4 at early times (left) and for the entire time course (right). The open squares are the experimental plasma ramiprilat values.


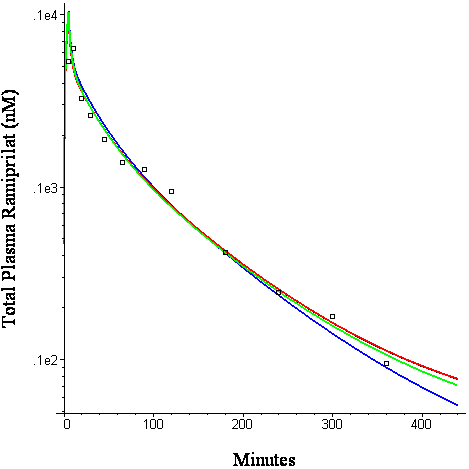

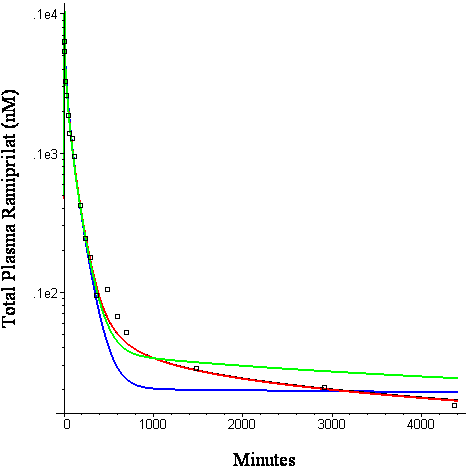


**“Standard” k-N = 0.0237/min; k-C= 0.00168/min**

**k-N = k-C= infinite**

**Reduce k-N and k-C by 0.01**

**Reduce k-N and k-C by 0.001**

**Subject 4: IV Ramiprilat Input**

Figure S4. Effect of variations in the plasma ACE concentration on the PBPK model plasma ramiprilat following IV ramiprilat for subject 4 at early times (left) and for the entire time course (right). Since the tissue/plasma ACE ratio is maintained constant, increases plasma ACE increases tissue ACE by the same fraction. The open squares are the experimental plasma ramiprilat values.


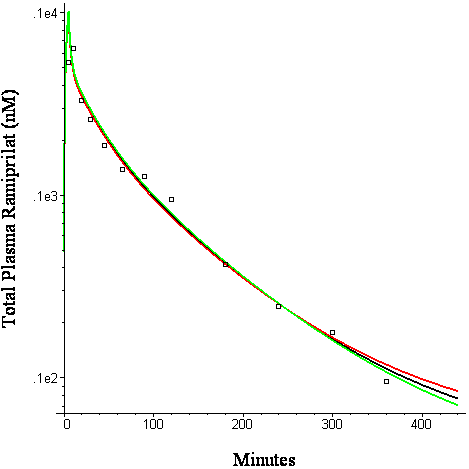

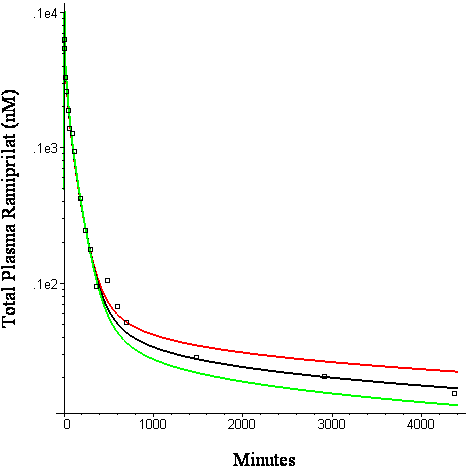


**Plasma ACE = 4.125 nM**

**Plasma ACE = 3.3** **nM**

**Plasma ACE = 2.64 nM**

**Subject 4: IV Ramiprilat Input**

Figure S5. Effect of variations in the tissue/plasma ACE concentration ratio on the PBPK model plasma ramiprilat following IV ramiprilat for subject 4 at early times (left) and for the entire time course (right). The plasma ACE is unchanged and the tissue ACE changes by the same fraction in all tissues. The open squares are the experimental plasma ramiprilat values.


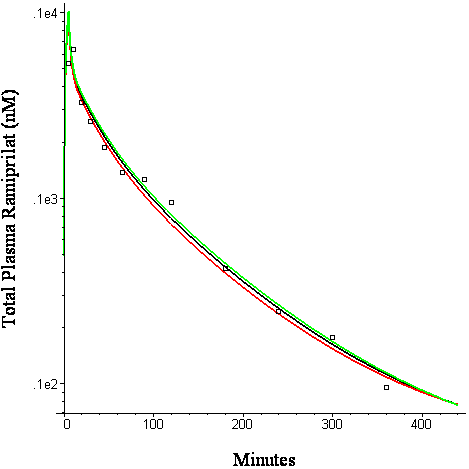

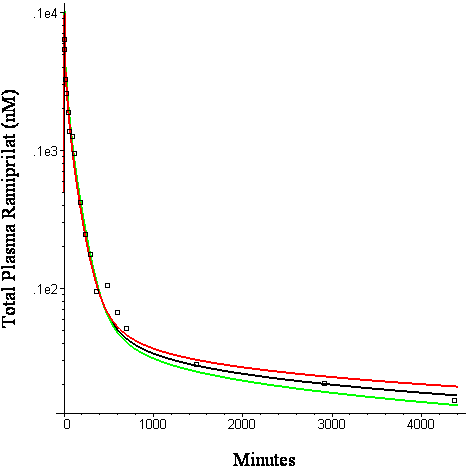


**Increase tissue ACE by 1.5**

**Standard tissue ACE**

**Decrease tissue ACE by 0.67**

**Subject 4: IV Ramiprilat Input**

Figure S6. Use of experimental ramiprilat renal clearance data following IV ramiprilat input to distinguish between different combinations of parameters for subject 4.


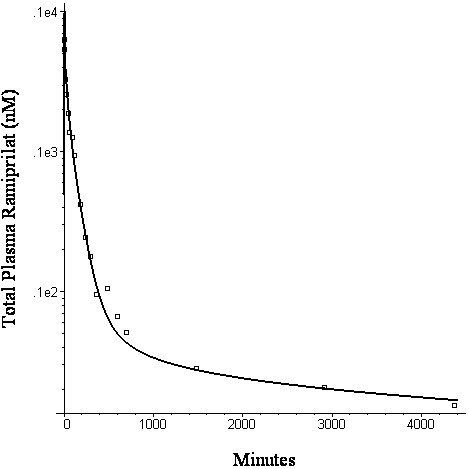

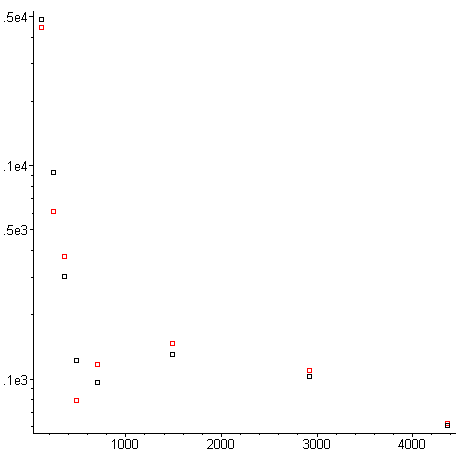

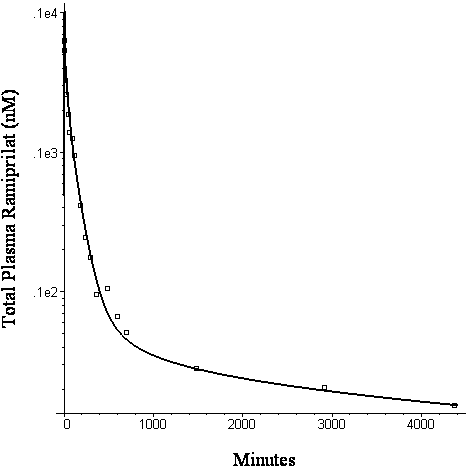

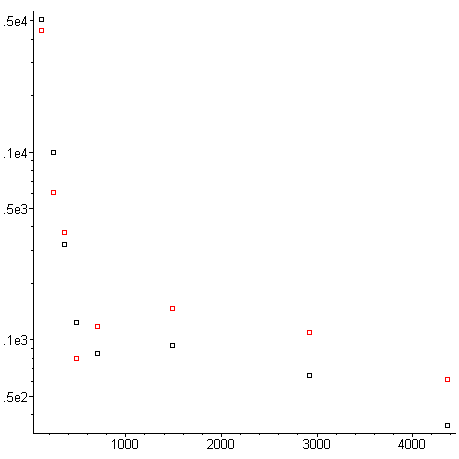

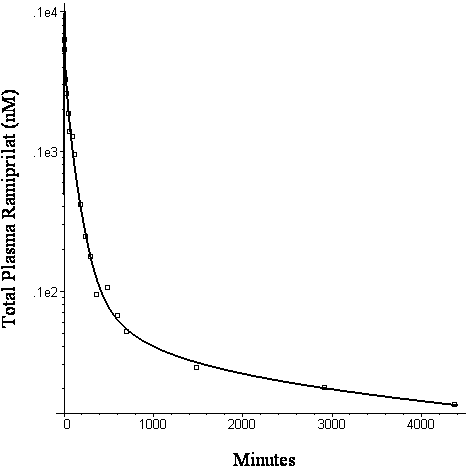

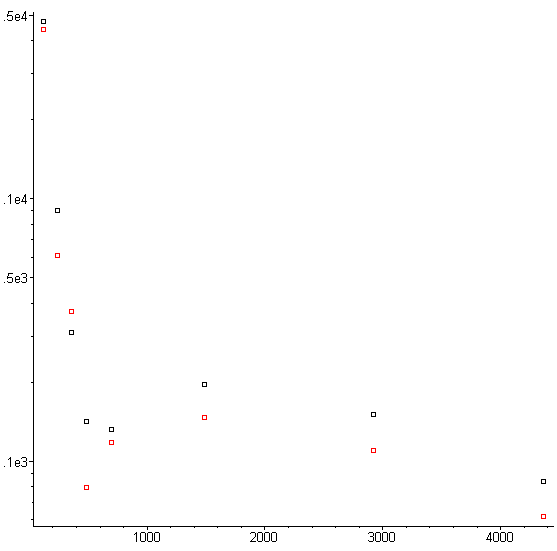


**Urine Ramiprilat (nm)**

**Urine Ramiprilat (nm)**

**Urine Ramiprilat (nm)**

**Standard Parameters**

**Plasma Ace = Standard x 1.25**

**Tissue Ace = Standard x 0.4**

**Plasma ACE = Standard x 1.25**

**KN an = Standard x 3.0**

**Renal Ramiprilat Clearance**

**PBPK model**

** Experimental**

Figure S7. Same plot as fig. 5 (main text) except no renal metabolism. The other parameters have been adjusted to optimize the fit to all the data.


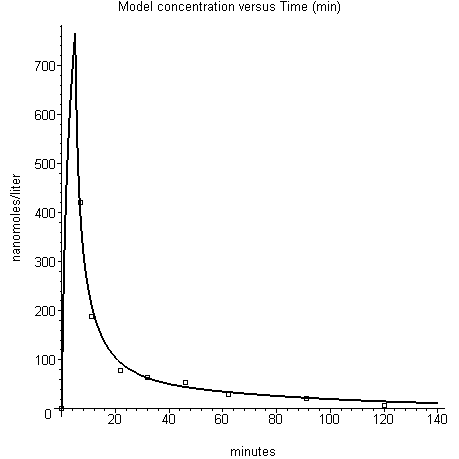

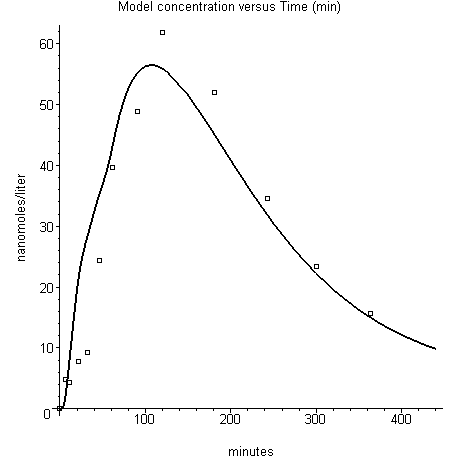

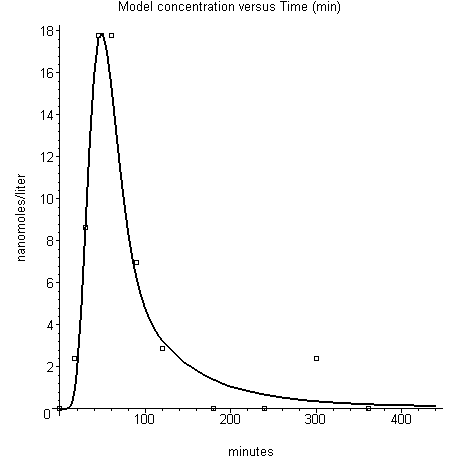

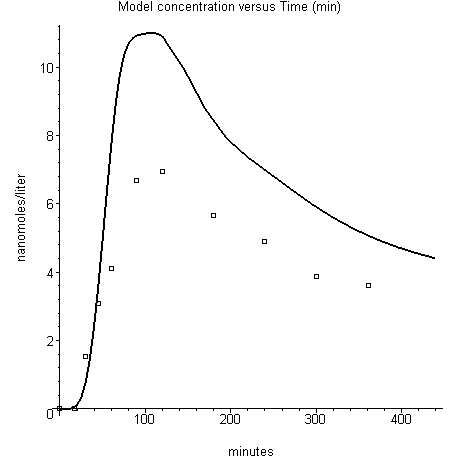


**Subject 4: No Kidney Metabolism**

**Plasma Ramiprilat (nM)**

**Plasma Ramiprilat (nM)**

**Plasma Ramipril (nM)**

**Plasma Ramipril (nM)**

**IV Ramipril**

**Oral Ramipril**

Figure S8. Effect of variations in the liver cell membrane ramiprilat permeability on the PBPK model plasma ramiprilat following oral ramipril for subject 4. The open squares are the experimental plasma ramiprilat values.


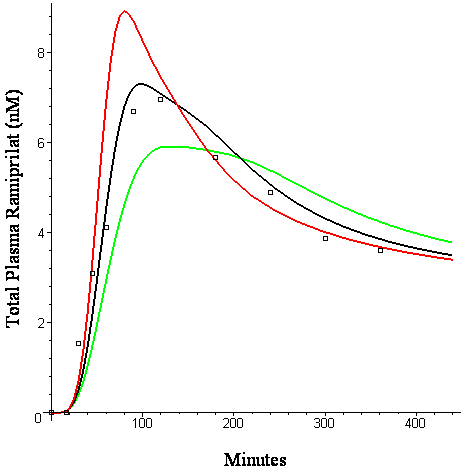


**Liver Ramiprilat Ps (min-1)**

**0.036**

**0.018**

**0.009**

**Subject 4 Oral Ramipril**

Figure S9. Effect of variations in the tissue (not liver or kidney) cell membrane ramipril permeability on the PBPK model plasma ramipril following oral ramipril for subject 4. The open squares are the experimental plasma ramipril values.


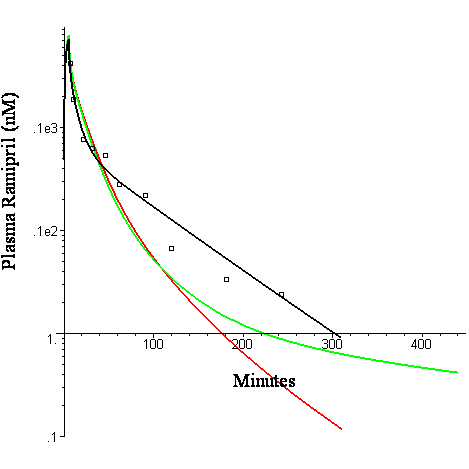


**Tissue Ramipril Ps (min-1)**

**0.0**

**0.005**

**0.05 to infinity**

**Subject 4: IV Ramipril**

Figure S10. Plasma ramipril (top) and ramiprilat (bottom) following oral ramipril for subject 4 with (black) and without (red) the slow intestinal ramipril absorption component.


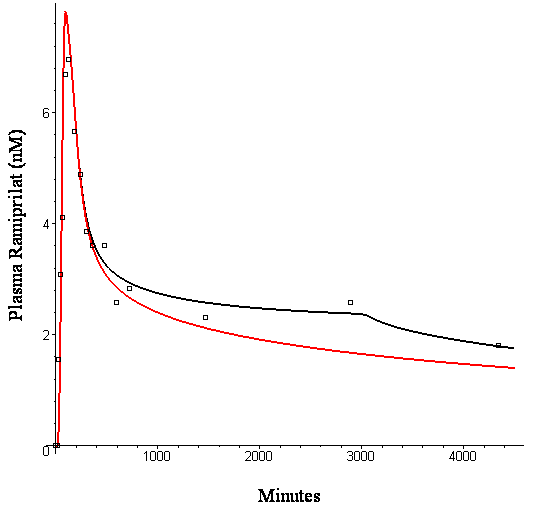

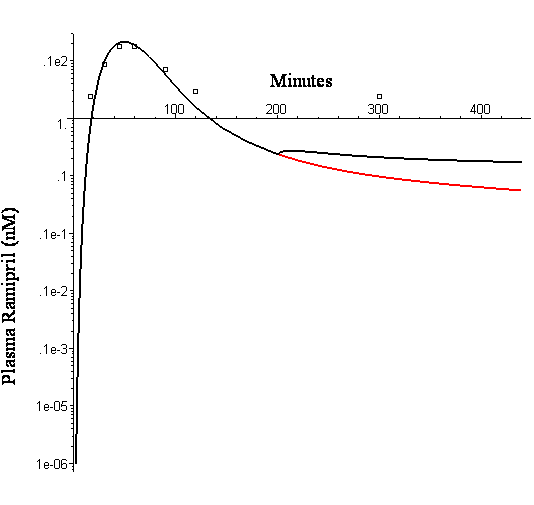


**Subject 4 Oral Ramipril**

**With slow component ramipril absorption**

**No slow component ramipril absorption**

Figure S11. Experimental data for subject 12. Top panels: plasma ramipril (left) and ramiprilat (right) following IV ramipril. Bottom panels: plasma ramipril (left) and ramiprilat (right) following oral ramipril. The peak in the plasma ramiprilat occurs earlier for the oral ramipril than for the bolus IV ramipril.


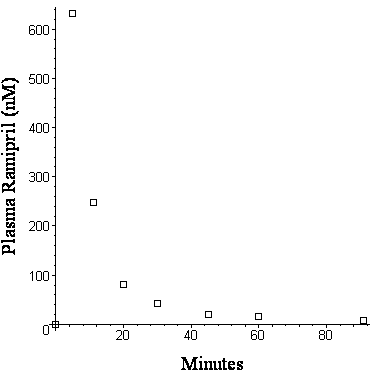

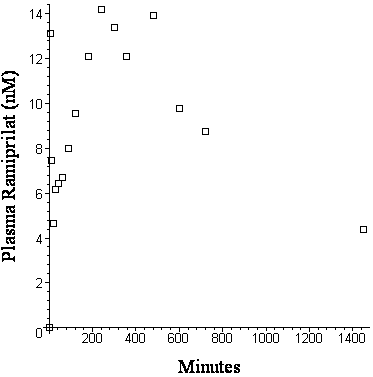

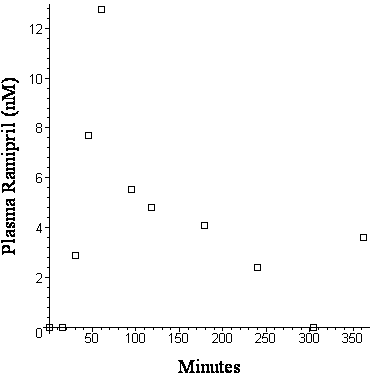

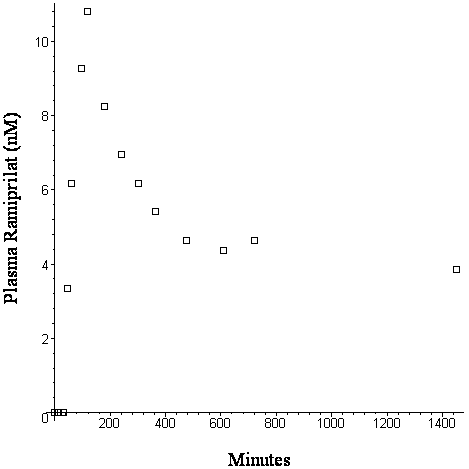


**Subject 12**

**IV Ramipril**

**Oral Ramipril**

Figure S12. PBPK model fit to the experimental data for oral ramipril for subject 12 with (black) and without (red) the direct ramiprilat absorption component.


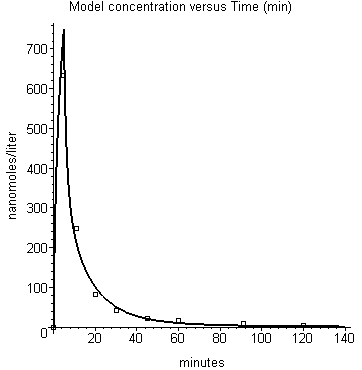

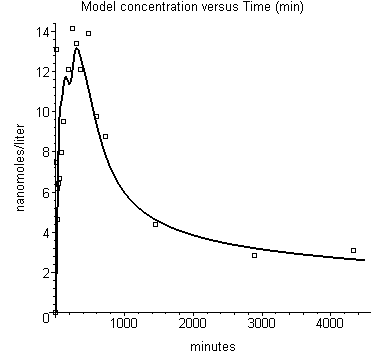

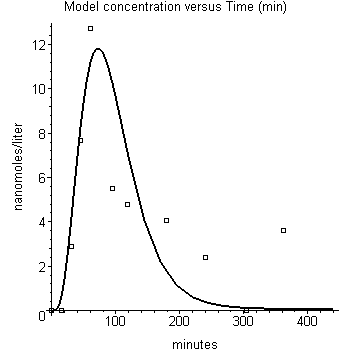

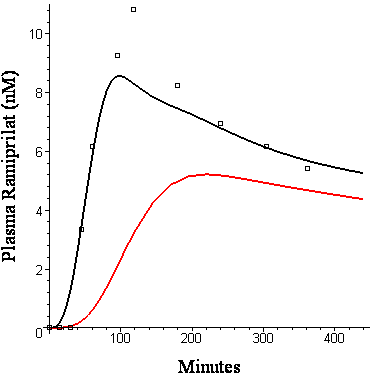


**Subject 12**

**IV Ramipril**

**Oral Ramipril**

**Plasma Ramiprilat (nM)**

**Plasma Ramipril (nM)**

**Plasma Ramipril (nM)**

Figure S13. Effect of variations in the ramiprilat dissociation rate constant for the N and C site on the plasma ramiprilat following oral ramipril in subject 4.


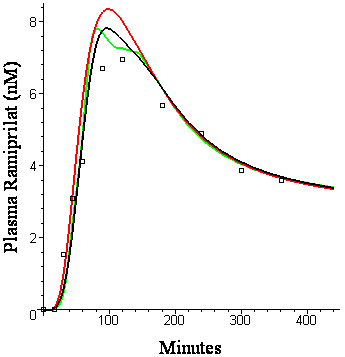


**Subject 4: Oral Ramipril Input**

**“Standard” k-N = 0.0237/min; k-C= 0.00168/min**

**Reduce k-N and k-C by 0.1**

**Increase k-N and k-C by 10**
